# Microbial applications for sustainable space exploration beyond low Earth orbit

**DOI:** 10.1038/s41526-023-00285-0

**Published:** 2023-06-21

**Authors:** Allison P. Koehle, Stephanie L. Brumwell, Emily P. Seto, Anne M. Lynch, Camilla Urbaniak

**Affiliations:** 1 Department of Plant Science, Pennsylvania State University, University Park, PA USA; 2 Department of Biochemistry, Schulich School of Medicine and Dentistry, The University of Western Ontario, London, ON Canada; 3 Honeybee Robotics, Altadena, CA USA; 4 Department of Pulmonary Medicine, The University of Texas MD Anderson Cancer Center, Houston, TX USA; 5 Graduate Program in Developmental Biology, Baylor College of Medicine, Houston, TX USA; 6 ZIN Technologies Inc, Middleburg Heights, OH USA; 7 NASA Jet Propulsion Laboratory, California Institute of Technology, Pasadena, CA USA

**Keywords:** Microbiology, Biotechnology, Biological techniques

## Abstract

With the construction of the International Space Station, humans have been continuously living and working in space for 22 years. Microbial studies in space and other extreme environments on Earth have shown the ability for bacteria and fungi to adapt and change compared to “normal” conditions. Some of these changes, like biofilm formation, can impact astronaut health and spacecraft integrity in a negative way, while others, such as a propensity for plastic degradation, can promote self-sufficiency and sustainability in space. With the next era of space exploration upon us, which will see crewed missions to the Moon and Mars in the next 10 years, incorporating microbiology research into planning, decision-making, and mission design will be paramount to ensuring success of these long-duration missions. These can include astronaut microbiome studies to protect against infections, immune system dysfunction and bone deterioration, or biological in situ resource utilization (bISRU) studies that incorporate microbes to act as radiation shields, create electricity and establish robust plant habitats for fresh food and recycling of waste. In this review, information will be presented on the beneficial use of microbes in bioregenerative life support systems, their applicability to bISRU, and their capability to be genetically engineered for biotechnological space applications. In addition, we discuss the negative effect microbes and microbial communities may have on long-duration space travel and provide mitigation strategies to reduce their impact. Utilizing the benefits of microbes, while understanding their limitations, will help us explore deeper into space and develop sustainable human habitats on the Moon, Mars and beyond.

## Introduction

The National Aeronautics and Space Administration (NASA) has pledged to return humans to the Moon in the next two years and land the first humans on Mars by 2033. The journey beyond low Earth orbit (LEO) will expand human civilization, enable future space settlements, provide scientific knowledge of the evolution of our planet and the solar system, and create global partnerships in the quest for further space exploration^1,2^. Under the Artemis plan, a crewed lunar flyby is scheduled for 2024 (Artemis II), followed by a lunar landing in 2025 (Artemis III)—the first since the end of the Apollo era in 1972, and eventually a sustainable lunar presence by the end of this decade^3^. Critical to the success of the Artemis program will be Gateway, an orbiting platform where astronauts will live and conduct research, while providing support for lengthy expeditions on the lunar surface. The Artemis program will establish a base camp at the lunar south pole that will serve as a steppingstone for human missions to Mars. Research and development at the lunar base will act as prototypes for these future Mars missions, where NASA can establish best practices for long-term human exploration in these adverse extraterrestrial environments^4^.

**Fig. 1:**
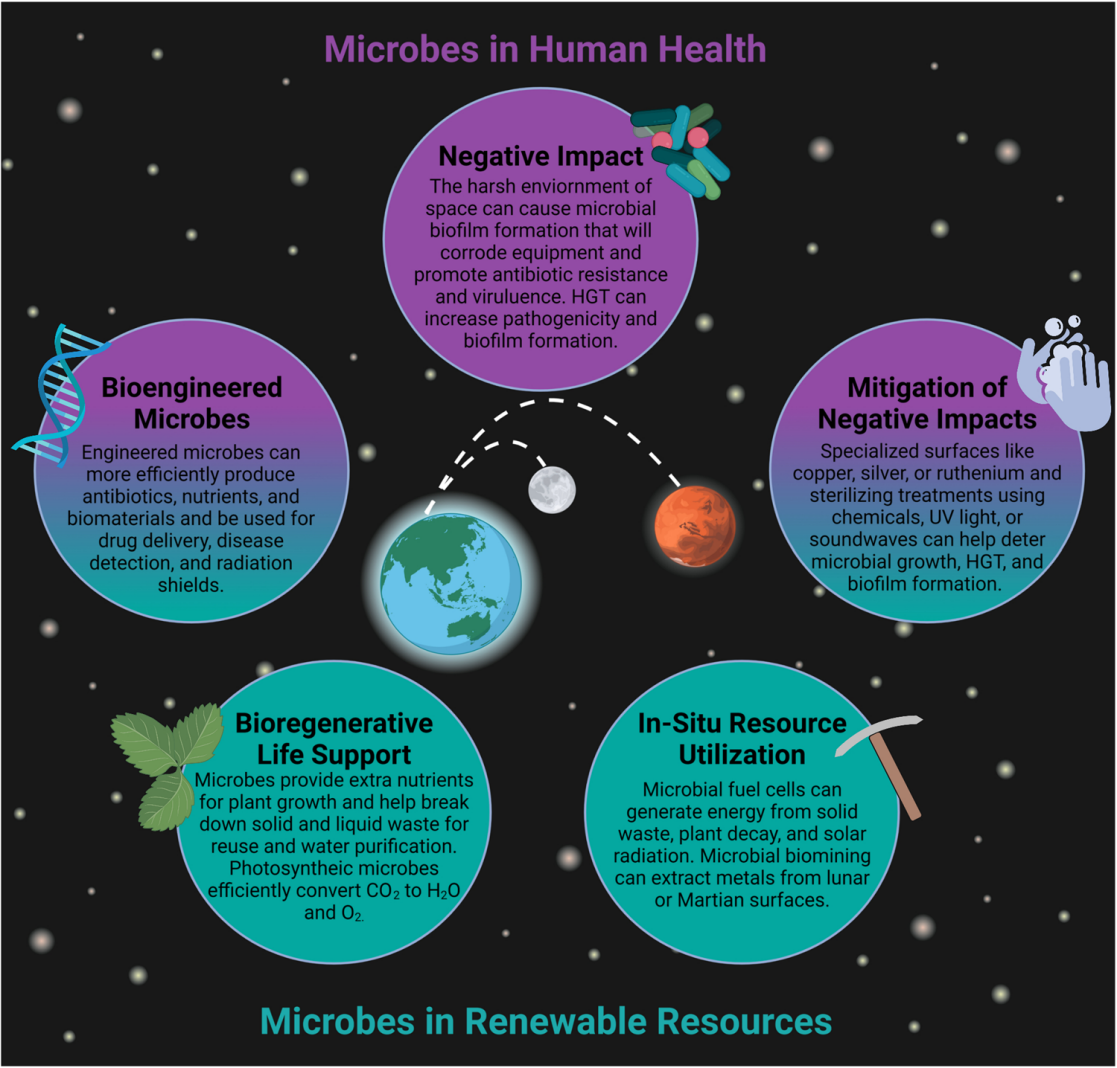
Summary of microbial impact during long-duration space missions. Space exploration can benefit from the use of microbes in a variety of applications including incorporation into biological life support systems (BLSS), in situ resource utilization beyond LEO, and astronaut therapeutics. However, increased pathogenicity and biofilm formation during spaceflight could threaten astronaut health and spacecraft integrity so mitigation strategies will be needed to prevent such hazards. Microbial applications related to health (purple), renewable resources (green) or both (purple and green) are highlighted. Figure created with BioRender.com.

Unlike the operation of the International Space Station (ISS), which is regularly resupplied from Earth within hours after launch, deep space missions will require self-sufficiency and sustainability independent of Earth. This will involve utilization of renewable resources, recycling of waste, power generation, and a continuous supply of food, water, and oxygen over a prolonged/indefinite period. The moon is the shortest distance beyond LEO with a deep space environment offering unique research opportunities to be conducted under the Artemis program. The lunar orbiter Gateway will function similarly to the ISS utilizing a Power and Propulsion Element that will use solar energy to propel and power the spacecraft, a Habitation and Logistics Outpost that will serve as the living quarters and research workspace, and docking ports for spacecraft such as Orion, that will be the first of its kind to transport astronauts to and from deep space^5,6^. The ISS and Earth-orbiting satellites capitalize on solar energy as a renewable resource for power, however in more distant outposts such as Mars, other factors like distance from the sun, angle, and weather (i.e., dust storms) affect the efficiency of energy provided by the solar arrays^7^. Such was the case with NASA’s Insight mission, where a recent Martian dust storm led to accumulated dust on the solar panels preventing adequate sunlight from reaching them, forcing the lander into battery-conserving “safe mode”^8^. Similar dust coverage issues were experienced during Apollo missions due to electrically charged lunar dust adhering to solar panels on the lunar lander^9,10^. Resupply cargo, like those that are frequently sent to the ISS, is costly, and may not be feasible for long-duration space missions (it takes ~7 months to get to Mars). Thus, self-sustainability in food and oxygen production on extraterrestrial outposts, such as on the Moon and Mars, is crucial^11^. In addition, communication delays between Earth and Mars can range from 5 to 20 min depending on the position of the planets^12^. Lack of cargo resupply missions and communication delays can be detrimental to human health-related emergencies making it imperative for crew members to be self-sufficient in health risk prevention and treatment. Therefore, solutions to address limited resources and human health risks that can be feasibly implemented in deep space must be established prior to the Artemis and Mars exploration missions. This could be achieved through the exploitation and engineering of microbes important to human health^13–16^, agriculture^17^, food production^18–20^, the ecosystem^21–25^, and the built environment^26,27^. Figure 1 provides an overview of the various roles microbes could play in deep space exploration.

In this review, we will examine some key considerations for planning crewed space missions that allow for self-sufficiency and sustainability and specifically the role that microbes can play in achieving these goals. We will also discuss the possible detrimental effects of microbes that could derail a mission, such as biofouling and increased pathogenicity, and suggest mitigation strategies to help alleviate some of these concerns.

## Bioregenerative life support systems and the value of microbial inclusion

NASA has been sending astronauts to space for the last 60 years, and with the advent of deep space missions to the Moon and Mars, astronauts must be self-sufficient to provide atmospheric conditions and necessities for human life (i.e., purified water and nutrient-rich food)^28,29^. This self-sufficiency can be achieved by implementing bioregenerative life support systems (BLSS). BLSS generate essential resources for human survival through biological processes, with four main purposes: higher plant cultivation, water treatment, solid waste bioconversion, and atmosphere revitalization. Microbes play a vital role in these biological processes by reducing the storage and resupply of materials necessary for a life-sustaining, regenerative environment.

Research on BLSS dates back to as early as the 1960s, by researchers worldwide. Examples include NASA’s “BioHome,” a 650 sq ft closed system that utilized a wetland system for wastewater recycling, and a biological system including plants and microorganisms for reducing organic contamination from wastewater^29^; the Soviet space program’s Biosphere 3 (BIOS-3), an underground closed system of phytotrons, that consists of a crew area and an entirely enclosed greenhouse, growing wheat and vegetables as well as algae cultivators for air revitalization^30^; the European Space Agency’s (ESA) Micro-Ecological Life Support System Alternative (MELiSSA), which includes five compartments where plants and microorganisms purify the air, produce food, and recycle waste^31^; and Beighan University’s Lunar Permanent Astrobase Life-support Artificial Closed Ecosystem (Palace) 1, comprising three cabins that work simultaneously to manage atmospheric conditions, produce crops, breed insects, and recover solid and liquid waste^32^. In all these support systems, microbes are an essential component in the regulation, degradation and circulation of materials and energy, ultimately enhancing the effectiveness of these life support systems.

### Plant cultivation

Research and development of higher plant cultivation, a method of growing crops with increased productivity, enhanced nutritional value, higher volume utilization, and shorter production cycle, are necessary for the development of sustainable ecosystems in space. Higher plant cultivation modules within BLSS not only provide a means for food production but also the recycling and revitalization of air through photosynthesis, and water recycling through transpiration and waste management^33^. Because of their importance, plant modules, and the effects of extraterrestrial conditions on plant growth have been extensively studied^34–36^. For example, NASA is heavily involved in this area of research with its Advanced Plant Habitat (APH) and Vegetable Production System (Veggie) experiments on the ISS. Both APH and Veggie are automated growth chambers used by researchers to determine the effects of microgravity on plant gene expression, protein, and metabolite levels, as well as their nutritional value^37^. The Veggie system has successfully grown lettuce, Chinese cabbage, mizuna mustard, red Russian kale, and zinnia flowers on the ISS^37^ and has enhanced our understanding of plant-microbe-environment interactions in microgravity^38^. Using the Veggie system, Hummerick et al. characterized microorganisms found on the leaves of three leafy greens: red romaine lettuce, mizuna mustard, and green leaf lettuce, as well as the microbial communities associated with the rhizosphere in the pillow component^39^. Characterization revealed higher microbial diversity near roots and within root substrate compared to leaves, consistent with plants grown in terrestrial soils. Molecular and culture-based methods revealed that the crops were pathogen-free and safe for human consumption. The information gained from the APH and Veggie experiments, especially as it pertains to plant-microbe interactions, provide a foundation for future research on higher plant cultivation in microgravity and the expansion of these ideas for plant production on extraterrestrial outposts.

One issue with hydroponic growth systems on Earth is microbial contamination, most often by *Fusarium oxysporum*^40^. Certain abiotic conditions such as high humidity, high temperature, and reduced airflow can cause undesirably high levels of microbial growth^41,42^. Veggie is a hydroponic system that has also succumbed to these limitations. *Zinnia hybrida*, an annual flowering plant, grown in the Veggie system on the ISS, developed foliar, stem, and root rot disease, due to high water stress and low airflow^43^. Whole genome sequencing analysis of the diseased tissue^44^ and subsequent virulence assays^43^, identified the culprit as *F. oxysporum*. This illustrates the potential difficulties of growing crops in hydroponic systems, on Earth or in space.

An alternative to a hydroponic system is a soil-based one where Martian and lunar regolith can be used as an alternative to terrestrial soil. This in situ resource utilization (ISRU) of regolith would reduce the need for costly resupply missions of terrestrial soil. While many plants and crops have been successfully grown in simulated Martian and lunar regolith their characteristics do differ from what would be expected with terrestrial soil^45–49^. One limiting factor of regolith is the absence of reactive nitrogen, an essential nutrient for optimal plant growth and function^50–54^. However, the introduction of nitrogen-fixing and nitrogen-cycling bacterial species into regolith to bind nitrogen from the atmosphere and transform it into reactive nitrogen (in the form of NO3− and NH4+) could be used as a method to improve regolith soil fertility^55^.

Increased Martian soil fertility through symbiotic relationships has been examined with clover (*Melilotus officinalis*), grown in simulated regolith that had been inoculated with the nitrogen-fixing bacterium, *Sinorhizobium meliloti*^56^. This study found that after three months, inoculated treatments produced greater clover biomass compared to uninoculated treatments, 0.29 g and 0.01 g, respectively. However, when *S. meliloti* inoculated clover was grown in common terrestrial potting mix the total clover biomass was seven-fold greater than when grown in simulated regolith^56^. While plant-bacterial symbiosis could improve soil fertility and plant growth in Martian regolith, additional experiments are required to achieve terrestrial levels of plant biomass.

Other plant stressors, such as limited nutrients, may prevent plants from reaching optimal biomass^56^. Essential nutrients, such as potassium, calcium, magnesium, iron, manganese, nickel, and zinc, are present in extraterrestrial soils but not at sufficient levels for plant uptake. Zaets et al. showed that bacteria can increase the bioavailability of these minerals in simulated regolith using inoculants of *Pseudomonas sp*. IMBG163, *Pseudomonas aureofaciens* IMBG164, *Stenotrophomonas maltophilia* IMBG147, *Paenibacillus sp*. IMBG156, *Klebsiella oxytoca* IMBG26, and *Pantoea agglomerans* IMV^45^. When inoculated with these bacteria, increased bioavailability of essential nutrients in the soil and plant tissue of *Tagetes patula* (i.e., French marigold) led to increased plant growth, seed germination and survival^45^. Conversely, only 20–30% of plants grown in non-inoculated soils achieved seed germination^45^. In addition to increasing nutrient bioavailability, these bacteria were also able to reduce toxic levels of zinc, chromium, nickel, iron, calcium, and sodium, by up to 50%, within plant tissue^45^. By increasing nutrient availability and reducing toxic accumulation of ions within the soil, microorganisms can be used as a tool for conditioning Martian and lunar basalt for effective plant growth and plant nutrient uptake.

Water is another crucial plant resource limited on both the Moon and Mars. Previous discoveries have found evidence of liquid water flows on Mars, coming from giant ice slabs beneath the surface^57^, though extracting and recycling water from these ice slabs is energetically impractical. In addition, Martian soil has limited water-holding capacity due to low organic carbon content, however, this can be improved by using bacteria that produce polysaccharides or adhesive proteins that bind soil particles, thereby increasing the moisture content of soil^58^. This microbe-soil interaction can be exploited on Martian outposts to reduce the need for copious amounts of water, increase soil stability, and prevent soil desiccation. Several studies on agricultural soils show that the application of microalgae and cyanobacteria to the soil can improve soil fertility and health^59–61^. Nascimento et al. assessed the ability of the N-fixing cyanobacteria *Nostoc sp*. to act as an organic fertilizer and soil conditioner under normal and drought conditions^60^. Researchers applied *Nostoc sp*. and urea as liquid fertilizers to soil growing wheat (*Triticum aestivum*), corn (*Zea mays*), and common bean (*Phaseolus vulgaris*). Drought conditions were simulated by watering the plant to water holding capacity and drying the soil for 14–16 days. Under drought conditions, plants fertilized with *Nostoc sp*. reached a biomass 150% greater than plants continuously watered to water holding capacity; while plants fertilized only with urea attained only 70% of the biomass compared to those continuously held at water holding capacity. Researchers also found that untreated soils exhibited more leaf wilting from water stress compared to those plants grown in soil treated with *Nostoc sp*^60^. This research shows the promise that cyanobacteria can have for improving soil quality for plant growth beyond LEO.

While Martian and lunar regolith are promising soil sources, they contain heavy metals, such as lead, cadmium, chromium, and arsenic, that can negatively impact plant growth and soil microbial fitness^62,63^. Microbes can be used for bioremediation to convert Martian and lunar regolith into soil capable of plant growth^64–66^. Huang et al. tested the ability of *E. coli* and *B. subtilis* to remove lead, cadmium, and chromium by cultivating samples in solutions containing varying heavy metal concentrations and environmental conditions, including pH, temperature, and equilibration time. Researchers found that both microbes successfully removed heavy metals under all conditions, though under optimal conditions, *E. coli* removed 60–69% of cadmium, lead, and chromium while *B. subtilis* removed 54–70% of cadmium, lead, and chromium^67^. Plant-microorganism interactions can also be a source of bioremediation by using plant growth-promoting rhizobacteria that can simultaneously remove toxic heavy metals and improve crop growth and yield^68^. Henao and Ghneim-Herrera investigated this bioremediation method by summarizing results from over 85 research articles and found that *Acinetobacter*, *Agrobacterium*, *Arthrobacter, Bacillus*, *Enterobacter*, *Klebsiella*, *Mesorhizobium*, *Microbacterium*, *Pseudomonas*, *Rhizobium*, *Rhodococcus*, and *Variovorax* all exhibited resistance to heavy metals and a high potential for bioremediation. Specifically, *Klebsiella* and *Enterobacter* exhibited the highest tolerance to heavy metals in soil and the greatest potential to mitigate plant growth inhibition under high arsenic, cadmium, and lead concentrations^68^. These results are mirrored by Yetunde Mutiat et al. ^69^, who assessed the removal efficiency of heavy metals under varying pH levels by wild-type and mutant strains of *Klebisella varicola*. Isolated *Klebisella* strains were exposed to various concentrations of lead, cadmium, arsenic, and nickel, resulting in removal of cadmium under all conditions with a maximum removal efficiency of 97.9 and 99.4% at optimal conditions of pH 7 for both wild-type and mutant strains.

Microbes can also be used to remove toxins from Martian soil such as perchlorates, which are found in high levels in Martian soil and cause a significant reduction in plant survival and productivity^70,71^. Engineered CO_2_-utilizing bacteria expressing perchlorate reduction enzymes have been shown to remove harmful perchlorates from the soil while also adding essential nutrients into the soil, such as chloride ions, oxygen, and water for better plant growth^72–74^. Sunikumar et al. tested the ability of two perchlorate-reducing soil bacteria, *Pseudomonas stutzeri* and *Azospirillum brasilense*, to reduce perchlorates from simulated regolith and found that they removed up to 5 mM and 10 mM of perchlorates, respectively, which corresponded to a removal efficiency of 100%^75^. These results suggest that naturally occurring or genetically engineered microbes with high perchlorate and/or toxin-reducing efficiency should be further studied for bioremediation of perchlorate and other harmful toxins from Martian and lunar soils.

Just as microorganisms are a vital part of terrestrial plant production systems, microorganisms will play an important role in higher plant production and soil systems on future deep space missions and extraterrestrial outposts. Previous research indicates that plant production using hydroponic systems is a promising method for plant production in microgravity^34–36^, but further optimization will be required to prevent fungal contamination in these systems^43^. Using soil-based plant growth systems is a promising alternative to circumvent the limitations of hydroponics, but research is limited in this area within BLSS. Therefore, further research using soil-based plant growth systems, supplemented with microorganisms, may improve the effectiveness of BLSS and self-sufficiency of astronauts on deep space missions.

### Wastewater treatment

Water is the largest product consumed in bioregenerative systems, expending nearly 20 L per person per day^76^. Extensive water consumption results in large wastewater production, including urine and flush water, atmospheric condensate, sink, shower, laundry, and dish water. Microbes play a vital role in the recycling of wastewater and nutrients through recycling systems containing combinations of anaerobic digestion, distillation, and disinfectant units.

Microbes also play a crucial role in solid waste processing (including bodily waste), inedible plant material, and other solid decomposable substances within bioregenerative systems. Drying is the first step to recycling solid waste^30,32,77^. This step allows the extraction of water from solid waste, the retention of organic matter, and the removal of inorganic material^78^. Dried, solid waste is then fermented in a solid waste bioreactor containing microbes that degrade plant waste^32,79,80^. This method has shown solid waste degradation rates between 41% and 87.7%^79^. The degraded solid waste can either be taken out of the system or applied to a plant system, providing a carbon and nitrogen-rich source of residue fertilizer or soil-like substance that increases soil fertility and overall plant health and productivity^81–83^.

There are many proposed systems for microbe-assisted waste purification and recycling on spacecraft. The MELiSSA initiative proposed a loop of compartments that thoroughly recycle gas, liquid, and solid waste using microorganisms, where each output of the preceding compartment provides the input for the following compartment^84^. Compartment I is an anaerobic digester that utilizes thermophilic bacteria to break down inedible plant parts and solid and liquid waste. *Clostridium thermocellum* ferments cellulosic substrate, while *Clostridium thermosaccharolyticum* degrades starches and pectins, leaving volatile fatty acids, minerals and NH_4_^+^ as an output. In compartment II, photoheterotrophic bacteria, such as *Rhodospirillum rubrum*, metabolize volatile fatty acids. The remaining minerals and NH_4_^+^ enter compartment III where nitrifying bacteria, such as those in the species *Nitrobacter* or *Nitrosomonas*, nitrify NH_4_^+^ to NO_3_^−^, which can be utilized in the plant compartment as a fertilizer^84^. Overall, this system results in a nitrogen-rich output that can be utilized as fertilizer in the plant compartment for improved production.

Another system proposed by Tang et al. utilizes a two-system recycling unit for either domestic water or wastewater^79^. Domestic water is purified by first running it through a two-stage membrane bioreactor and then passing it through a nanofiltration system, to produce hygiene water. The second system utilizes anaerobic, mostly Bacteroidetes, and aerobic, mostly Proteobacteria, microbial bioreactors to recover organic matter and N from wastewater^79^. Within this system, microorganisms are also utilized to degrade solid waste as part of the microbial fermentation facility or Bio-toilet. The facility includes a source separation module that separates urine from feces, a primary bioreactor where feces are combined with other inedible plant material to be degraded by microorganisms, and a secondary bioreactor for further degradation by microbes. This system was tested during 108-day experiment housing four crew members at the China Astronaut Research and Training Center. Researchers achieved 100% water regeneration with 87.7% recycled solid waste^79,80^.

Although BLSS can obtain 100% water recovery, nitrogen recovery efficiency is still lacking. One option to improve nitrogen recovery is to utilize urease-producing microorganisms to hydrolyze urea, a compound found in human urine at high levels (>13 g/L)^85,86^. Urease-producing microorganisms, such as *Bacillus, Sporosarcina, Pseudomonas*, and *Paracoccus*, used in conjunction with membrane-biological activated carbon reactor systems by Xie et al. showed that BLSS can obtain water recovery of 100% with N recovery of up to 79.33%, which are comparable to efficiencies obtained by Tang et al.^79^. Another urine-fueled system for waste recycling, proposed by Maggi et al., includes a soil-based BLSS aimed at recycling liquid wastes using a plant-microbe system^87^. The growth chambers for dwarf wheat and soybean contain three systems for water and urine injection, atmospheric circulation, and ventilation. Once injected into the soil, a number of bacteria can release nitrogen-based intermediates, such as NH_4_^+^ and NO_3_^−^ from organic nitrogen compounds for plants to uptake. Results indicated that urine decomposition met the nutrient demands of the plants as evidenced by successful growth of the dwarf wheat and soybean plants with comparable biomass generation to those grown on Earth.

Plant-microbe systems can provide other methods of wastewater recycling. Plants are excellent water purifiers and can release 2–10 L of water vapor from their leaves through the process of transpiration^88^. Plants uptake water through their roots, absorb nutrients into plant tissue, and transpire water through their stomata. Applying wastewater as a means of watering plants would effectively turn wastewater into clean water through this natural process. However, before plants can be exposed to wastewater, it would need to be pre-treated to reduce organic loading in soil and remove phytotoxic or other detrimental compounds that would affect plant growth and metabolism^89,90^. This can be achieved with microbial bioreactors through the mechanisms described above, allowing for eco-friendly water reclamation.

### Atmosphere revitalization

It is projected that crew members on a lunar mission will inhale about 1 kg of O_2_ per day and exhale approximately 1.3 kg of CO_2_^91^. Production of O_2_ and removal of CO_2_ during space missions could be achieved through photosynthesis, the process by which plants, algae and cyanobacteria convert CO_2_, sunlight, and water, into O_2_ and energy^92^. Cyanobacteria are the earliest oxygenic photosynthetic organisms on Earth and have been contributing to Earth’s atmospheric oxygen for the last 2.5 billion years^93,94^. One advantage of using cyanobacteria over plants for air revitalization is their ability to perform photosynthesis with far less sunlight than is required for plant growth. Under normal conditions, plants and cyanobacteria use chlorophyll-*a* to convert visible (i.e. “white”) light into energy, but some cyanobacteria can perform far-red photosynthesis, using chlorophyll-*f*, a spectrally red-shifted variant of chlorophyll-*a* which absorbs longer wavelengths of light^95–97^. This allows those cyanobacteria to also perform photosynthesis and harvest energy when grown in low- or filtered- light environments^95–97^. This photosynthetic efficiency, coupled with the ability to survive the harsh conditions of space^98–101^ make cyanobacteria ideal components in BLSS destined for the Moon and Mars.

Photobioreactors can be incorporated into BLSS to increase the production of oxygen by cyanobacteria or algae for enhanced air revitalization. ESA’s MELiSSA project is a BLSS concept focused on the regeneration of atmospheric gases and water, waste treatment, and food production for crewed space missions^102,103^. The system comprises the listed compartments, each with a specific organism contributing to the recycling pathway^104^. One of the five compartments includes a gas-lift photobioreactor containing photosynthetic cyanobacteria, specifically *Spirulina platensis*, that uses the CO_2_ produced by its predecessor compartment to produce oxygen^84^. *S. platensis* was chosen for its light energy conversion efficiency, its ability to tolerate fluctuations in pH, and its high nutritional value (containing 55–70% protein, 15–25% carbohydrates, 18% essential fatty acids in addition to vitamins, minerals, and pigments^105^). Another species of cyanobacteria that is being considered for air revitalization, nitrate removal and edible biomass production in MELiSSA is *Limnospira indica*. In a recent 35-day ground study, *L. indica* was grown in a simplified closed-loop version of MELiSSA and the effect of urea, ammonium (the prominent nitrogen forms present in non-nitrified urine) and nitrate, on the oxygen production capacity of *L. indica*, was measured^106^. It was observed that cyanobacteria fed nitrate or urea could effectively reach the desired (set point) O_2_ level of 20.3% and maintain ambient O_2_ levels, while those fed ammonium could only reach a maximum O_2_ level of 19.5%^106^. This study provided preliminary evidence for the use of ammonium-rich and urea-rich media (such as urine), for *L. indica* cultivation and air revitalization. *L. indica* has also been grown in photobioreactors on the ISS, as part of the Arthrospira-B spaceflight experiment, and no inhibitory effect on oxygen production and growth was observed, as compared to ground controls^107^.

These studies show the promise of cyanobacteria-based BLSS and/or photobioreactors destined for the Moon and Mars to provide clean air for crew in spacecraft or in lunar/Mars habitats. Additional research is needed for optimization such as identifying additional candidate species, growing combinations of different cyanobacteria for synergistic effects, and testing more growth conditions to achieve enhanced biomass and increased efficiency.

## Biological in situ resource utilization for sustainability

In addition to BLSS which can increase self-sufficiency and sustainability beyond LEO, the ability to utilize in situ resources, will also play a role in long-term human habitats on the Moon and Mars. For instance, electricity and power can be generated with microbial fuel cells (MFC) coupled with in situ organic material, and biomining can be used to extract resources for construction, repair, and maintenance of structural components and equipment.

### Microbial fuel cells

Microbial production of energy has gained much interest in the last decade. To keep pace with human energy consumption, many scientists have turned towards the use of microbial fuel cells as a sustainable method of energy production on Earth^108^. These alternative methods of energy production could also be applied for space exploration as a sustainable method to power the spacecraft, mission controls, and various life support systems.

**Fig. 2:**
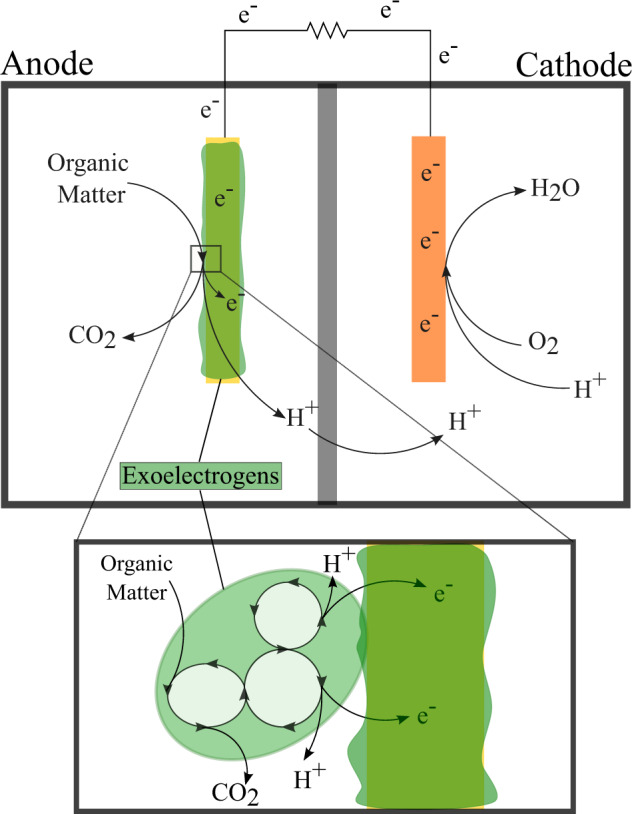
Microbial fuel cell. MFC consists of two chambers, separated by a proton exchange membrane. In the anode chamber, exoelectrogens, shown as a biofilm in this figure, anaerobically oxidize organic matter releasing protons and electrons. A closer look at this interaction can be seen in the bottom image. The electrons released during the oxidation of organic matter transfer to the anode and travel to the cathode in the second chamber via an external circuit, creating an electrical current. The protons released travel through the proton exchange membrane into the cathode chamber, where the protons and electrons react with oxygen and form water.

MFC are small, lightweight devices that convert organic matter from renewable sources into electricity using microorganisms as catalysts^109^ (Fig. 2). Microorganisms involved in this electrochemical activity are called exoelectrogens because of their ability to transfer electrons exogenously to electron acceptors^109^. Some examples of exoelectrogens include *Pseudomonas*^110^, *Shewanella*^111^, *Geobacter*^112^, and *Desulfuromonas*^113^.

The idea behind MFC has been around for over a century, but it is just within the past few decades that it has become a commercialized product. MFC can produce an energy output up to 5.61 W/m^2^^114–116^^,^ and can also be used for wastewater recycling, toxin removal, bioremediation, and resource recovery^117–121^. These same concepts can be utilized on future Martian or lunar extraterrestrial outposts for energy production and within BLSS. In 2007, de Vet and Rutgers were the first to test the capabilities of MFC energy production under simulated and real microgravity conditions aboard the ISS using *Rhodoferax ferrireducens* to produce electricity. Energy output averaged 0.1 mA in 1 G, 0.35 mA in simulated microgravity, and 0.02 mA on the ISS. While the differences were not statistically significant, the study did show the potential for MFC to operate in space^122^. This mechanism for energy production is not yet practical for space travel due to the low energy output, considering a standard 40 W light bulb draws 0.36 A to operate, but can be initially utilized for its byproducts, such as clean wastewater^123^.

#### Waste recycling as an energy source

Waste can accumulate during space travel in the form of urine, fecal matter, and inedible food and with the help of microbes, this waste can be repurposed for energy production as well as for nutrient recovery and production of potable water. Urine is an excellent feedstock for MFC as it contains high levels of urea, organic ammonium salts, and other organic compounds that microbes can convert into electricity^86^ thus making urine MFC effective mechanisms for energy production^124–127^. Some urine MFC can not only produce energy but recover nutrients as well. Lu et al. designed a three-chamber MFC to remove organic pollutants, recover N, phosphorus (P), and sulfur (S), and produce energy from urine^125^. The maximum power output was 1300 mW/m^2^, with almost complete removal of pollutants, including over 97% of urea, total nitrogen, sulfate, phosphate, and chemical oxygen demand, as well as 40% of ammonium, 15% of salts, and 91-99% of organic compounds. The MFC also recovered essential nutrients, including 42% of total N, 37% of phosphate, 59% of sulfate and 33% of total salts^125^. This recovery technology can be especially valuable within other compartments of BLSS, including plant compartments, by providing nutrient-rich water free of contaminants.

In order for urine MFC to also be used as a mechanism to convert urine to potable water, the high level of inorganic salts present in urine (~14.2 g/L)^86^ need to first be removed for MFC to function efficiently^125^. This can be achieved with an alternative type of MFC, called a microbial desalination cell, which follows the same concept as a standard MFC but with an added desalination chamber between the anode and cathode^128^. Cao et al. tested this mechanism of water desalination at concentrations comparable to the salinity of urine at 5, 20, and 35 g/L using a mixed bacterial culture, with the salt concentration determined by a change in conductivity of the solution^128^. This microbial desalination cell produced a maximum power output of 2 W/m^2^, with ~88–94% of salt removed, depending on the initial concentration^128^.

Other organic components of wastewater, such as human feces, can be a resource for electricity generation by MFC as well. Fangzhou et al. tested the capabilities of MFC to generate electricity from activated sludge obtained from a sewage treatment plant for specific use within BLSS for future crewed outposts^129^. Tests were performed using a standard or adjustable two-chamber MFC, a one-chamber MFC with one or two membrane electrode assemblies, and a fermentation pre-treatment device. The highest maximum power output was 70.8 mW/m^2^ produced by the two-chamber MFC, however, the authors concluded that for space applications, the one-chamber configuration was better, as it produced a more stable output, at 0.3 V^129^. The efficiency of pollutant removal was also tested, with about 44% removal of ammonium and 71% of organic material with each configuration^129^. To further increase power generation and toxin removal from fecal wastewater, fermentation pre-treatment was proposed. This involved using reactors filled with anaerobic sludge to degrade fecal macromolecules into smaller organic molecules^129^. Pre-treating fecal wastewater by fermentation produced 47% more power than no pre-treatment, suggesting a preference of exoelectrogens within MFC for smaller organic molecules^129^. Based on these results, the authors developed an automatic human feces wastewater MFC system containing a fermentation pre-treatment device to simultaneously dispose of one day’s worth of feces and generate electricity. Indeed, the maximum power output of the system was 240 mW/m^2^, about 3.5-fold higher than the standard two-chamber MFC system^129^.

Inedible food waste will be an inevitable part of spaceflight and extraterrestrial outposts on the Moon and Mars that need to be disposed of, as on Earth. This organic material can act as substrates in MFC for energy production, Colombo et al. tested the energy producing capabilities of MFC with various food-industry organic wastes as inputs, including those rich in fibers, sugars, proteins, and acid^130^. A one-chamber MFC was fed each type of organic substrate, and the concentration of organic compounds was measured periodically to obtain the rate of degradation. The maximum power output for each organic waste substrate was 50 mV for sugar, 40 mV for fiber, 30 mV for protein, and 10 mV for acid, with each organic compound degraded by 90%^130^.

While MFC will be a useful tool to create energy and recycle organic waste beyond LEO, research and development is still ongoing to develop more efficient systems with a larger and sustained power output. Some of these ideas involve the use of different materials (such as ceramics) and configurations (large vs small, stacked vs dispersed)^131^. Gajda et al. tested a small (70 mm long, 15 mm diameter, 2 mm thickness) and a large (100 mm long, 42 mm diameter, 3 mm thickness) terracotta MFC. They found that the smaller terracotta MFC achieved a power density output 2.9-fold greater than the large MFC, at 20.4 W/m^3^ and 7.0 W/m^3^, respectively. Gajda et al. also tested the performance of stacking MFC for a small-scale multi-unit system that could be utilized on future crewed outposts^132^. They compared power output of a small module containing 28 MFC units and a larger module containing 560 MFC units. Stacked 560 units created a five-fold improvement in power output of 245 mW compared to the 28 MFC unit. Another concept is the PeePower urinals which collect urine and feces directly from the source, producing energy through multiple ceramic MFC^133^. This leads to concentrated wastewater inputted into the MFC rather than diluted samples, which reduces power output. Researchers tested a 288-unit MFC on a university campus which averaged 5–10 users per day. The PeePower urinals were able to produce an average of 75 mW which powered the LED lights directly connected to the MFC stack for 75 h. Another 432-unit MFC was tested during a large music festival which averaged 1000 users per day. In this setting, the PeePower urinals were able to produce an average of 300 mW which successfully powered lighting within the urinals over a seven-day period^133^. While the success of PeePower was demonstrated on Earth, it will be important to test similar models of power generation using urine and feces in microgravity. None the less, this research provides the foundation for the development of similar toilet-like MFC to be used for power generation on deep space missions.

#### Plant MFC

Plant compartments within BLSS can be used for energy production in MFC as well. Healthy soils contain organic matter from decaying plant litter as well as carbohydrate flux directed out of the roots into the rhizosphere^134^. In theory, the anode chamber of a MFC could be positioned within the rhizosphere to capitalize on the symbiotic microbes present to oxidize this continuous source of organic matter to generate an electrical current. Such a soil MFC was tested using rice plants, where 330 W/ha of power was produced in the presence of actively growing plants, a seven-fold higher energy output compared to the energy output of soil MFC not using plants^135^. This technology is not limited to only soil-based systems but can be applied to hydroponic plant systems as well, in which the anode is situated within the water chamber surrounding plant roots^136^. Research by Lee and Miller, growing *Bacopa monnieri* and with the addition of *Escherichia coli*, obtained a power density output of up to 1.9 W/m^2^ with a 34% increase in plant growth fueled by plant essential nutrients supplied by *E. coli* acting within the fuel cell^136^. In addition to electricity generation, soil MFC can be used for the remediation of heavy metal contaminated regolith^64^. Habibul et al. tested the ability of soil MFC to remove chromium from soil using ryegrass. The soil MFC was fed a solution of varying concentrations of chromium, resulting in >90% removal efficiency by Proteobacteria and Firmicutes. In addition, the higher the concentration of chromium, the higher the current density output, reaching a maximum of 55 mA/m^2^^137^. These results show the promise of energy generation through plant-system powered MFC with the added benefit of increasing plant yield for consumption by crewmembers.

#### Solar power

Photosynthetic microorganisms, such as algae or cyanobacteria, can be utilized to convert light energy into electrical energy, termed microbial electrochemical technology^72^. Biophotovoltaics is a specific type of electrochemical technology in which phototrophic microorganisms produce electricity by utilizing incoming light energy to split water molecules, generating electrons and protons that can be used to produce an electrical current within an MFC. Several cyanobacteria species have been tested for use in biophotovoltaics, such as *Synechocystis*^138,139^*, Nostoc*^140,141^*, Lyngbya*^142,143^, and *Leptolyngbia*^144,145^. Kaushik et al. tested the energy producing capabilities of *Synechococcus* using a two-chamber photosynthetic MFC built with light transparent glass^146^. The MFC operated through a 12-h light/12-h dark cycle under a white light intensity of 15 W/m^2^. Maximum power density output of the photosynthetic MFC was 0.61 W/m^2^^146^. This technology provides a feasible method of energy production on extraterrestrial outposts, but further research needs to be completed to increase power output and optimize light conversion.

Research on the use of in situ resources such as wastewater, plant systems, and solar radiation, shows potential for the use of MFC as a mode of power generation and sustainability on extraterrestrial outposts. Though power generation is limited from these substrates at the moment future work may enhance their efficiency. In addition, other sources of power, such as nuclear power, could supplement these MFC systems to provide adequate power generation in habitats and spacecraft beyond LEO^147^.

### Biomining

Biomining is an environmentally friendly and affordable alternative to traditional physical-chemical mineral processing methods to extract metals of economic interest from rock ores or mine waste. The process involves specific microorganisms that secrete organic acids and metal-binding compounds that essentially dissolve these metals, allowing them to be easily extracted from the environment^148^. Biomining is commonly applied to pyritic ores and completed by iron-oxidizing bacteria, such as *Thiobacillus ferrooxidans*^149^, *Leptospirillum ferrooxidans*^150^, and *Acidimicrobium ferrooxidans*^151^. With the reduced iron in the form of pyrite, the bacteria produce iron that oxidizes metal sulfides to sulfuric acid which further accelerates rock dissolution^152–154^. These species, along with those in the *Sulfobacillus* and *Acidianus* genera, as well as many iron-oxidizing bacteria, are used for the biomining of copper, zinc, uranium, nickel, aluminum, and cobalt^155^.

The biomining process is not limited to Earth. It may serve as an innovative method for reducing the cost of raw materials and energy requirements beyond LEO, enhancing the sustainability of life on extraterrestrial outposts. Martian and lunar basalt are known to contain many valuable metals, such as iron, nickel, copper, vanadium, and many others, that are suitable substrates that can be biomined by microbes^156,157^. Biomining of these metals from Martian and lunar surfaces could provide the necessary materials for the in-situ construction of buildings, electrical systems, spacecraft equipment, solar cells, and heating and lighting systems in human habitats beyond LEO^158^.

Recent research on the ISS simulating biomining of essential compounds from basalt under microgravity demonstrated the possibility for microbial mining beyond Earth^159–161^. Cockell et al. tested the rare Earth element (REE) biomining capabilities of three microorganisms, *Sphingomonas desiccabilis, Bacillus subtilis, and Cupriavidus metallidurans*, under three different levels of gravity: microgravity, simulated Martian gravity, and terrestrial gravity, and against a non-biological control^160^. Biomining reactions took place within biomining reactors. Within each reactor, researchers placed growth media, sterilized basalt slides with a known REE and single strain cultures of each microorganism. Biomining capabilities were assessed based on absolute quantities of REE in ng obtained from 6 mL bulk fluid collected from the biomining reactors and compared to the non-biological control, consisting of a sterile basalt slide without cell inoculation^160^. REEs assessed include lanthanum, cerium, praseodymium, neodymium, samarium, europium, gadolinium, terbium, dysprosium, holmium, erbium, thulium, ytterbium, and lutetium. The concentration of each REE extracted was proportional to the known abundance in the basaltic rock. At all simulated gravity levels, *S. desiccabilis* demonstrated enhanced biomining capabilities per gram of basalt substrate, producing 32.52 ng under microgravity, 43.09 ng under Mars gravity, and 32.26 ng under Earth’s gravity, compared to the non-biological mining control, which produced 24.67 ng under microgravity, 21.36 ng under Mars gravity, and 13.25 ng under Earth’s gravity. These values represent the combined mass of biomined REEs. *B. subtilis* and *C. metallidurans* demonstrated no differences under the simulated gravity conditions tested and underperformed compared to the non-biological control. As part of the same flight experiment, Cockell et al. tested the biomining capabilities for vanadium (a critical, high-strength element used as a building material), using the same methods and organisms as the Cockell et al. study described above^160,161^. *S. desiccabilis* and *B. subtilis* increased mined vanadium yield, achieving a two-fold increase in mined vanadium 184.92% and 283.22% under microgravity, 216.32% and 219.78% under Mars gravity, and 208.70% and 221.59% under Earth’s gravity, respectively, compared to the control^160^.

With the abundance of iron in Mars regolith (17.9% wt), iron may be a crucial resource produced through biomining^162^. Iron is one of the most-processed metals on Earth that is incorporated in most building materials and would be heavily relied on for construction, repair, and maintenance of buildings at extraterrestrial outposts. Copper is another important metal that can be produced through biomining, with nearly 20–30% of all copper produced on Earth extracted through biomining^162^. For over 30 years, copper has been an essential metal used in the construction of rocket engines^163,164^ and being able to extract copper and other minerals from in situ resources on extraterrestrial outposts will allow engine maintenance and repair to occur beyond LEO, reducing the cost and time of sending replacement parts from Earth.

Other economically essential elements have been found in asteroidal material and Martian regolith and can be extracted through biomining^165–167^. These include those in the platinum group, including palladium and osmium, and the 17 REEs. During the Viking Mission to Mars, palladium-silver tubing was utilized in gas chromatography-mass spectrometry to detect organic compounds, and it would be important for future research on Mars in the search for extraterrestrial life^168^. In addition to machinery, REE can be used in building and fixing methods for power generation, specifically solar panels^169^. Lastly, REE are found in electronic screens and fluorescent lights, both necessary for data collection, communication, and the general well-being of those on extraterrestrial outposts^170^.

#### The biomining process

**Fig. 3:**
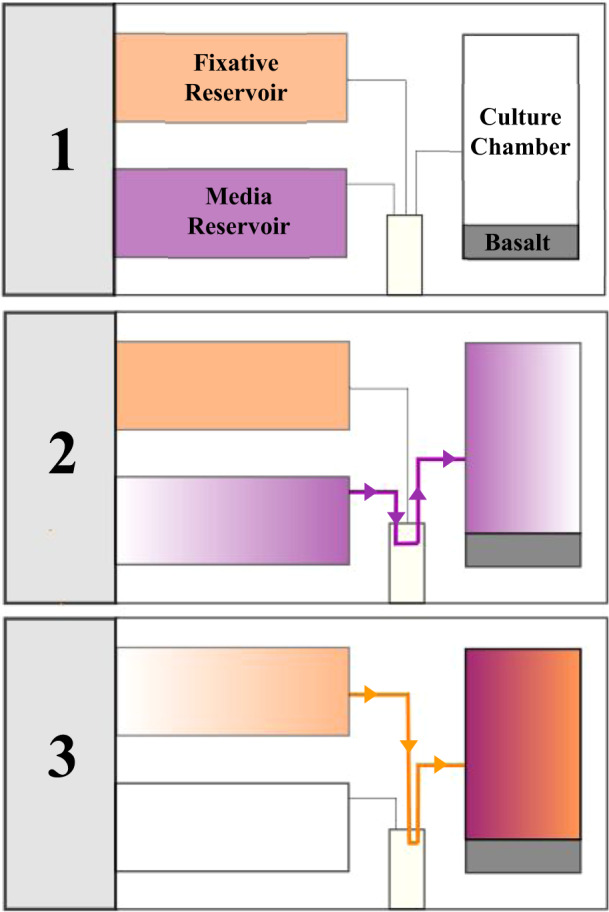
Schematic depiction of a biomining reactor. The biomining reactor has three main components: the culture chamber, the media reservoir chamber, and a fixative reservoir chamber (shown in section 1). The culture chamber is where the biomining reactions take place and where the microorganisms reside before the media is injected. The media reservoir chamber contains the nutrients required for the biomining reaction to occur and is injected into the culture chamber to begin the biomining reaction (section 2). Once the biomining reaction is completed, a fixative is injected to halt microbial growth (section 3).

Bioreactors are necessary for biomining reactions to occur. Terrestrial biomining processes most often occur in open, non-sterile tank reactors that require constant stirring to distribute oxygen and nutrients^171^. To implement biomining on extraterrestrial outposts, it is essential to assess the extent to which differing gravity levels impact microbe-mineral interactions within these stirred-tank bioreactors. An experiment called BioRock, aimed to do this by creating a prototype biomining reactor for space experimentation on the ISS^159^. The biomining reactor has three main components: the culture chamber, the medium reservoir chamber, and a fixative reservoir chamber, where a fixative is injected to halt microbial growth after the biomining reactions take place (Fig. 3). Two biomining reactors are placed together within two levels of containment. Pre-test flights found the bioreactors to be successful at growing the model microorganisms, *S. desiccabilis, B. subtilis*, and *C. metallidurans*. These microorganisms were chosen as they are low-risk pathogens with the ability to survive desiccation for space flight, limited requirements for growth, and are present in mineral-rich environments. Growth was determined based on optical density in nutrient solution after three weeks. For *S. desiccabilis*, growth occurred in all tested geometries of biomining reactors, ranging from 0.308 to 0.804 OD^159^. BioRock has also been successfully used to test REE and vanadium biomining capabilities of *S. desiccabilis, B. subtilis*, and *C. metallidurans* in microgravity, Mars gravity and Earth’s gravity^160,161^.

An additional method of biomining, proposed by Volger et al., utilizes a two-bioreactor system and aims to further enhance ISRU on Martian outposts compared to traditional bioreactors^172^. The first system is an algae bioreactor, which utilizes *Chlorella vulgaris* to produce biomass for the biomining reactor and oxygen. The algal biomass is then utilized by *Shewanella oneidensis* as a growth medium in the biomining reactor. In the biomining reactor, *S. oneidensis* mines iron ores from Martian regolith; the biomass-rich material left over after extraction can then be used for plant growth. Based on modeled algae growth and biomining performance, the system is projected to produce 0.031 kg O_2_ per day and 100 kg of iron per Mars year^172^. This model needs to be further tested and future experiments should include exposure to various gravity conditions to assess the impact that this spaceflight stressor will have on growth and performance.

The BioRock experiment and other biomining endeavors using iron-oxidizing and alternative candidate bacteria demonstrate potential for biomining in differing gravity levels and the potential for biomining as a source of ISRU at future Martian and lunar outposts. Biomining for elements known to be located within Martian and lunar regolith, such as iron and REE, will be essential for proper maintenance and production of devices and technology that promote sustainability and provide a foundation by which to launch operations for deep space exploration.

## Bioengineered microbes for space

Microorganisms are an important, renewable resource that can be leveraged to produce pharmaceuticals or therapeutics, biological life support systems, and manufacturing materials for human space exploration and colonization that could help reduce the need for costly resupply missions beyond LEO^173^. Candidate microbes can be chosen for these applications based on the availability of genetic tools for manipulation, desired metabolic properties, and tolerance to environmental conditions. These microbes can be further engineered to make them more well-suited for biotechnological applications for interplanetary travel or extraterrestrial settlements using synthetic biology tools. Synthetic biology involves the rational design or repurposing of living organisms and biological systems. Using synthetic biology, microorganisms can be engineered or built de novo with characterized parts and tools to endow them with new or improved functions^173^.

### Biotherapeutics

The risk that long-duration space missions pose for crewmembers is not yet completely understood but the extreme conditions, such as microgravity, radiation, and confinement, coupled with microbiome dysregulation may lead to or enhance the disruption of bodily functions^174^. Researchers have studied the effect of simulated or actual spaceflight conditions on gastrointestinal (GI) problems^175^, the development of diseases such as cancer and cardiovascular disorders^176^, or a predisposition to contracting infections^177–179^. The use of probiotics as a countermeasure to combat changes in the microbiome as a result of spaceflight is being investigated to support astronaut health on long-duration space missions^180,181^. Probiotics are living organisms able to survive in the gastric environment that provide health benefits and maintain or improve microbiome balance when consumed. On Earth, probiotics have been used to treat many ailments including weight and muscle loss, inflammation, dermatitis, immune disorders, mental health, and GI conditions (i.e., diarrhea, irritable bowel syndrome (IBS), inflammatory bowel disease (IBD))^180,182^.

#### Probiotic viability in space

Promising probiotic candidates for space missions could include *Bifidobacterium* and *Lactobacillus*, to counteract their decrease in relative abundance in the astronaut microbiome during spaceflight^183,184^. While these species are commercially used on Earth their efficacy and long-term viability when used and stored in space has to still be verified. In 2017, Shao et al. examined the viability of *Lactobacillus acidophilus* in simulated microgravity and observed no effect on cellular morphology or adhesion. However, some biological changes were present compared to controls, such as increased growth rate at early time points, acid tolerance (pH < 2.5) by ~22–32%, bile tolerance at low concentrations, antibacterial activity, and resistance to antibiotics (i.e., cefalexin, gentamicin, penicillin)^185^. The following year, the shelf life of freeze-dried *Lactobacillus casei* strain Shirota press-through capsules was tested in spaceflight conditions aboard the ISS^186^. After one month of storage in ambient conditions (i.e., temperature 20–24.5 °C, absorbed dose rate 0.26 mGy/day) and six months after the start of the experiment, bacteria in flight samples were sustained in sufficient numbers that were comparable to ground controls. There were no observed changes in probiotic viability, and the basic probiotic properties of the bacteria including growth rate, carbohydrate fermentation, cell-wall polysaccharide integrity, and resistance to intracellular digestion remained intact upon thawing^186^. A lengthier shelf-life analysis of freeze-dried cells for three commercial probiotics including *Bifidobacterium longum, L. acidophilus* and spores of *B. subtilis* was then performed in a simulated three-year round-trip to Mars^187^. In under 200 days, *B. longum* and *L. acidophilus* viability was decreased by about 2-logs while *B. subtilis* maintained viability up to the end of the experiment (545 days). Therefore, researchers concluded that freeze-dried bacterial spores showed the most promise for withstanding long-duration space missions including ambient spacecraft conditions and radiation with an estimated shelf-life of 4.7 years^187^. Overall, these studies provide foundational information on the storage, stability, and viability of probiotic candidates when flown in space. These results suggest that with further testing, probiotic bacteria can be an essential component of the astronaut medical toolkit for the maintenance of a healthy gut microbiome, prevention and treatment of bacterial infections or medical concerns that may arise in future space missions.

#### Engineered probiotics to combat infection

In addition to the observed decrease in beneficial bacteria, microbial tracking studies have shown that spaceflight conditions can also lead to an increase in opportunistic pathogens in both the built microbiome and astronaut microbiome. This is particularly problematic due to the dysregulated immunity of astronauts in space^178^. Since antibiotics are the most commonly used therapeutic for the treatment of bacterial infections, researchers are investigating whether microbes can be used for antibiotic production in space. This is particularly relevant considering that antibiotics are known to have accelerated degradation and decreased efficacy when flown and stored in space for long periods of time^188^. On the Space Shuttle Mission STS-77, Lam et al. analyzed the effects of spaceflight on the production of monorden by *Humicola fuscoatra* WC5157, a marine fungus. Monorden has demonstrated antimicrobial activity against pathogenic fungi and antitumour activity on human tumor cell growth in vitro^189^. Using solid-state fermentation, researchers observed up to 190% increased yield of the antifungal in spaceflight compared to ground controls at 23.8 and 8.2 μg, respectively^190^. Similar results were obtained in another study analyzing the production of actinomycin D, an antibiotic and antitumor agent, by *Streptomyces plicatus* on the ISS. After 17 days in orbit, the amount of antibiotic produced by ISS samples increased by up to 577% compared to ground controls^191^, and over a 72-day period, researchers noted increased production of actinomycin D in ISS samples specifically at early time points^192^. While much work is yet to be done toward on-demand microbial production of antibiotics in space, these studies demonstrate that microbes are a promising platform for this application. Elucidating the mechanism driving this increased yield early in antibiotic production in microgravity could enable the engineering of bacteria for enhanced antibiotic or therapeutic bioprocessing in space or even be applied to increase antibiotic production on Earth.

Antibiotic-associated diarrhea (AAD) may arise in space as a side effect of administering antibiotics to treat infections. Several studies have investigated the use of *Debaryomyces hansenii* as a treatment for AAD in a mouse model. *D. hansenii* is commonly used in the food industry for the processing of cheese and has been identified as part of the human gut microbiome^193,194^. When administered for treatment of AAD, *D. hansenii* alters the composition of the microbiome by promoting the growth of beneficial lactase-producing bacteria and by inhibiting the growth of opportunistic pathogens^195–197^. In one study, the presence of Proteobacteria in the intestinal mucosa increased in response to diarrhea, from ~19% in a normal group to ~36% in an AAD model group, and treatment with *D. hansenii* was able to restore Proteobacteria to normal levels^195^. Proteobacteria abundance often positively correlates with IBD and inflammation and is generally regarded as an indicator of microbiome instability^198^. In spaceflight, the proportion of Proteobacteria in the astronaut skin microbiome was decreased, namely Gammaproteobacteria and Betaproteobacteria^175^, while it was increased in the salivary microbiome^199^.

While bacterial infections are typically treated with antimicrobials, the formation of biofilms and propagation of multi-drug resistance in the spaceflight environment (discussed later in this review) limits these therapeutic options. A promising alternative currently being investigated on Earth is the use of engineered microorganisms as live biotherapeutics (e.g., biosensors, probiotics with enhanced benefits, and drug delivery systems)^200–204^. Well-established microbial chassis organisms include *E. coli* and *Saccharomyces cerevisiae*, though a microbe more well-suited to the target therapeutic environment or application can also be chosen. One example using a less conventional chassis was recently performed by Garrido et al. where they engineered *Mycoplasma pneumoniae*, a human lung pathogen, as a live biotherapeutic to treat *S. aureus* and other biofilm-associated infections in vivo^205^. Researchers created an attenuated strain of *M. pneumoniae* able to secrete anti-biofilm and bactericidal enzymes, dispersin B and lysostaphin, and demonstrated its ability to eliminate an *S. aureus* biofilm in a mouse model^205^. Another candidate chassis that could be employed to eradicate pathogens is *D. hansenii*, which produces volatile organic compounds and mycocins that have demonstrated antimicrobial effects on several pathogenic bacteria and fungi. An alternative to secreting bactericidal proteins to kill pathogens is the incorporation of CRISPR/Cas9 gene-editing technology into synthetic designs to create engineered probiotics for targeted bacterial killing. This was demonstrated by Neil et al. by delivering CRISPR/Cas9 on a conjugative plasmid which led to 99.9% eradication of antibiotic resistant *E. coli* and complete eradication of *Citrobacter rodentium* in the GI tract of a mouse model^206^. CRISPR/Cas9 gene editing has also been demonstrated in *D. hansenii*^207^, and could be applied to engineer this strain for increased production of mycocins that can target *C. albicans*^208,209^.

Microbes can also be engineered as biosensors to identify or inhibit pathogenic bacteria by sensing an important indicator molecule and releasing a signal or enzyme in response. For instance, biological targeting systems have been demonstrated using engineered *E. coli* for directed killing of the biofilm-associated pathogen *Pseudomonas aeruginosa*, a bacterium that has been demonstrated to have increased biofilm formation and pathogenicity on the ISS^210^. Saeidi et al. engineered *E. coli* with a synthetic genetic circuit containing three modules: sensing, lysing, and killing. The sensing module includes a constitutively expressed transcription factor, *lasR*, which detects and binds to *N*-Acyl homoserine lactone (AHL), a quorum sensing molecule released from *P. aeruginosa*. This bound complex activates the lysing and killing modules expressing lysis E7 and pyocin S5 proteins, respectively. This leads to perforation of the *E. coli* cell membrane and release of the bacteriocin which targets the pathogen and killed 99% of viable cells^211^. Following this study, Hwang et al. programmed *E. coli* with a modular circuit containing the same sensing module coupled to a motility and killing module. The motility module expressed the chemotaxis protein CheZ to initiate motility toward the pathogen, while the killing module produced antimicrobial and biofilm-degrading proteins, MccS and DNaseI. Viability of *P. aeruginosa* was examined and found that *E. coli* harboring both the motility and killing biosensor modules resulted in the killing of 60% of cells^210^. Biological sensors to detect and/or reduce pathogenic bacteria using traditional chassis such as engineered *Lactobacillus* or *E. coli* have also been demonstrated against intestinal *P. aeruginosa*^212^, vancomycin-resistant *Enterococcus*^213^*, Candida albicans*^214^, and *S. aureus*^215,216^. These studies highlight the vast potential of engineered microbes to sense and kill space microbiome-associated pathogens and disrupt biofilms. Since the choice of probiotics is both bacteria- and ailment-dependent^217^, the use of synthetic biology to create genetically engineered biotherapeutics with higher complexity and multiple functions (i.e., able to target multiple pathogens) is vital to minimize the amount of cargo on future space missions. The viability of these therapeutics for humans and their ability to function in the conditions of spaceflight still need to be investigated, but these examples help to shed light on what the next generation of engineered biotherapeutics could offer.

#### Engineered probiotics for disease prevention and detection

Engineered probiotics can also be a valuable tool for the prevention or detection of more serious health issues such as GI disorders, kidney stones, cancer, and cardiovascular disease (CVD), or the treatment of their associated symptoms. Space-induced changes in the gut microbiome observed in astronauts aboard the ISS by Voorhies et al. included an increase in *Parasutterella*, a bacteria associated with IBD^175^. In IBD pathology, purinergic receptors are activated by extracellular adenosine triphosphate (eATP) released by commensal gut bacteria and immune cells, promoting intestinal inflammation. Engineered yeast probiotics containing a human P2Y2 purinergic receptor have been developed for the treatment of IBD by responding to physiological eATP levels and secreting the eATP-degrading enzyme apyrase^218^. This probiotic was shown to be effective in a mouse model of IBD, decreasing intestinal inflammation and dysbiosis. Bacterial probiotics can also be engineered to detect gut inflammation by sensing tetrathionate, thiosulfate or nitric oxide^219,220^, or with programmable memory systems to detect and respond to an environmental stimulus^221^.

The risk of kidney stones due to bone decalcification, dehydration, or increased growth rate of calcium-depositing nanobacteria^222–224^ is increased in spaceflight, which could also be prevented or treated using probiotics. Calcium oxalate is the major component of kidney stones, therefore ideal probiotic bacteria are efficient in oxalate degradation, such as *Oxalobacter formigenes*^225^ and *B. subtilis*. *B. subtilis* strain 168 has been presented as a novel probiotic therapy as it has been shown to break down the oxalate in kidney stones in a *Drosophila* model^226^. The oxalate decarboxylase (OxDC) enzyme derived from *B. subtilis* can also be used to engineer other bacteria as probiotics for the treatment of kidney stones. The expression and subsequent purification of this enzyme in *E. coli* was able to reduce oxalate concentrations in a mouse model in urine and feces by 44% and 72%, respectively, compared to controls^227^. The OxDC gene was also introduced into *Lactobacillus plantarum* on a plasmid, leading to expression and secretion of this enzyme where it was able to degrade 70–77% of oxalate in vitro, and reduced oxalate as well as calcium, uric acid, creatinine, serum uric acid, and BUN/creatinine ratio in urine compared to controls in a rat model^228^.

Probiotics including *Lactobacillus* and *Bifidobacterium* can also be used to improve cardiovascular health by reducing weight, cholesterol, and adipose tissue while also preventing or attenuating injuries to the heart (e.g., heart failure, ischemia, cardiac hypertrophy)^229^. Using a rat model, *Lactobacillus rhamnosus* or a placebo was administered to subjects following coronary artery occlusion for a six-week duration. Compared to placebo controls, rats given the probiotic treatment showed attenuation of left ventricular hypertrophy, improved systolic and diastolic left ventricular function, and additional improvements up to six weeks after withdrawing treatment^230^. Other *Lactobacillus* species have had positive effects on CVD including *L. plantarum* which helped to reduce serum levels of leptin and fibrinogen, which are CVD risk factors^231^. These probiotics can also be genetically engineered to enhance their potential benefits. For instance, *E. coli* Nissle 1917 has been genetically engineered to produce N-acylphosphatidylethanolamines which, when administered to mice, led to decreased adiposity, insulin resistance and lipid accumulation in the liver^232^. This has important implications for astronauts as spaceflight can induce negative, aging-like effects on the cardiovascular system (i.e., decreased fitness, arterial stiffening, and insulin resistance) and radiation exposure has been well-characterized to increase the risk of developing radiation-induced cardiovascular disease (RICVD)^233,234^.

Crewmembers are at an elevated risk of cancer development due to radiation and other spaceflight factors^235–237^. As such, methods for cancer prevention and treatment are important to implement during deep space exploration and bacterial-mediated cancer therapies could be a promising approach Bacteria can be used naturally or engineered for cancer therapy to specifically target and colonize tumors, or as a drug delivery system for anticancer agents^238^. Many bacteria have been investigated for these applications including *Bifidobacterium*^239^, *E. coli*^240–242^, *Clostridium*^243,244^, *Salmonella*^245–249^, and *Streptococcus*^250^ species. A study using *E. coli* engineered with synthetic adhesins to target a tumor antigen in vivo found that lower doses of engineered *E. coli* were required to colonize tumors compared to wild-type controls^240^. Tumor targeting was also demonstrated in an attenuated strain of *Salmonella typhimurium* harboring a short hairpin RNA expression plasmid. It was engineered to target inhibin, a tumor marker, resulting in significant inhibition of colon cancer and melanoma growth in a mouse tumor model^247^. Bacteria can also be engineered to improve tumor and metastasis visualization within mammalian hosts, facilitating their use as diagnostic and therapeutic microbial agents. For example, *E. coli* was engineered to express an acoustic reporter gene allowing them to be imaged noninvasively in vivo and to produce protein-nanoparticle gas vesicles for targeted breast cancer therapy^242^. *E. coli* was also engineered with *lacZ*, encoding the *β-galactosidase* reporter, which can be easily detected in urine as an indicator of liver metastasis^251^.

Bacteria hold great potential for the development of easily modifiable biotherapeutics that could be invaluable for treatment or prevention of health issues during long-duration space missions. However, additional research and clinical validation is necessary before employing engineered bacteria as biotherapeutics for space-associated disorders and diseases.

### Life support and nutrition

BLSS can provide crew members with oxygen, food, and water, and will be imperative for long-duration space missions and for the establishment of sustainable human habitats on the Moon or Mars. Due to their diverse applications for spaceflight, microalgae and cyanobacteria are often studied for their incorporation in BLSS and photobioreactors. They produce oxygen, remove carbon dioxide from the environment and help with water purification^104,105,252,253^. These microbes are also edible allowing their biomass to provide nutritional and therapeutic benefits without the need for protein purification^35,254^.

#### Enhanced photosynthesis

Oxygen for astronauts on the ISS is currently transported in pressurized tanks from Earth or is produced using water onboard through electrolysis^255^. Therefore, the ability to improve carbon uptake and oxygen output using microbes on the ISS, for space travel and in future extraterrestrial habitats, is an essential step toward the creation of sustainable and self-sufficient systems. Oxygen production, CO_2_ capture, and photosynthetic capacity could be enhanced in BLSS using synthetic biology tools to address the bottleneck of photosynthesis: the carbon fixation cycle. Metabolic engineering of cyanobacteria can improve photosynthetic capacity as demonstrated by Berepiki et al. where expression of mammalian cytochrome P450 (CYP1A1) acting as an electron sink in *Synechococcus* PCC 7002 improved photosynthetic efficiency and increased electron flow rate by ~30%^256^. Using the same cytochrome P450 gene, Santos-Marino et al. engineered metabolic pathways for sucrose production and cytochrome P450 as a carbon and electron sink, respectively, into *Synechococcus elongatus*. Ultimately, this resulted in increased photosynthesis, and simultaneous expression of both sinks had an additive effect on photosystem I oxidation and photosystem II efficiency^257^. Another strategy to improve this cycle is by increasing the substrate concentration of ribulose-1,5-bisphosphate carboxylase-oxygenase (RuBisCo) to improve carbon uptake. In the cyanobacterium *Synechocystis* sp. PCC6803, one study found that genetic installation of additional bicarbonate transporters resulted in a 2-fold increase in carbon uptake and biomass^258^. Metabolic engineering of cyanobacteria can also be used to produce industrially relevant high-value chemicals and bioproducts such as biofuels. Some engineering efforts have been demonstrated in the model cyanobacteria species, *S. elongatus* PCC7942 and *Synechocystis* sp. PCC6803, to produce ethanol, ethylene, isobutyraldehyde, and isoprene^259–262^. The biotechnologically relevant bacteria, *Ralstonia eutropha* (i.e., *C. necator*) has also been engineered by Dogutan and Nocera to capture CO_2_ to produce biofuels and edible biomass, in an artificial photosynthetic cycle that is much more efficient than those that are naturally occurring^263^.

#### Microbial production of nutrients

In addition to oxygen, microalgae can provide a sufficient source of proteins, carbohydrates, fatty acids, minerals, and vitamins required for a balanced diet^264,265^. These nutritional outputs can be further enhanced using synthetic biology approaches. Genome editing technologies for these marine species have expanded over the last 20 years, namely due to improvements in DNA sequencing, manipulation techniques and availability of genomic information^266^. Some of these methods and technologies include DNA delivery via conjugation, the generation of auxotrophic strains, and DNA-free or plasmid-based genome editing (e.g., using CRISPR/Cas9)^267–270^. Therefore, it is possible to create cell factories using metabolic engineering to alter the composition or nutritional output of these species^271–273^. For example, mutagenesis and CRISPR/Cas9 gene editing technologies have been used to modify the biomass composition of the model algae species *Chlamydomonas reinhardtii*. Irradiated mutant strains have been generated with double the starch content compared to the wild-type strain through increased expression of phosphoglucomutase 1 (PGM1) and decreased expression of downstream enzymes in the glycolytic pathway^274^. *C. reinhardtii* has also been engineered to knock out the zeaxanthin epoxidase gene resulting in 47-fold increased production of the carotenoid zeaxanthin, which is important in the prevention of macular degeneration^275^, a concern facing astronauts during prolonged spaceflight^276^.

Synthetic biology approaches are also being used to genetically engineer microorganisms for the production and long-term storage of nutrients as part of NASA’s BioNutrients projects^277^. Nutrient production and storage on long-duration space missions is a challenge as they can degrade over time. Therefore, this project aims to develop a system for on-demand microbial production of micronutrients on the ISS, whereby packages of dehydrated, edible yeast can be hydrated and consumed. In the first segment of the project, BioNutrients-1, *Saccharomyces cerevisiae* and *S. boulardii* species were engineered to produce antioxidants with genes for zeaxanthin and beta-carotene biosynthesis pathways, respectively^277^. The *S. boulardii* genome was also engineered to stimulate increased trehalose stores and with tardigrade-derived cytosolic abundant heat soluble (CAHS) genes, both resulting in increased tolerance to desiccation. Along with these strains, several other edible microorganisms are being tested for their storage and survival in stasis packages in the ambient conditions of the space environment. These include yogurt-producing and milk-coagulating bacteria (*Lactobacillus delbrueckii subsp. bulgaricus, Streptococcus salivarius subsp. thermophilus, B. subtilis* and *Bacillus coagulans*), yeasts (*Kluyveromyces lactis* and *Komagataella phaffii Kurtzman*) and C1-utilizing bacteria (*Methylobacterium extorquens* and *C. necator)*. The production and stasis packages were delivered to the ISS and will be analyzed for growth and nutrient expression for a five-year duration. After 47 days, initial stasis package data showed no significant difference in the viability of bacteria stored on the ISS compared to ground controls^278^. These organisms are all attractive candidates for biological engineering to produce vitamins, therapeutics, or other useful enzymes for maintaining crew health. In addition to continued testing of the production of carotenoids, BioNutrients-2^279^ aims to further develop the bioproduction system from BioNutrients-1 by expanding the variety of probiotic products on the ISS to include yogurt and kefir and investigate the production of follistatin by the engineered yeast *K. lactis*^279^. The results of the BioNutrients project will provide invaluable information for the feasibility of using microbes as a platform for nutrient storage and production for long-term space travel.

### Engineered biomaterials

In-space manufacturing and development can be challenging as necessary materials and supplies are not readily available and currently need to be transported or resupplied from Earth. Microbes offer a solution as they can be reprogrammed for the production of biologically derived materials (i.e., bioplastics, nanomaterials)^280^ to generate useful components such as plastics, adhesives, composites, and rubbers for structural space applications.

#### Gel-based materials

One material that would be beneficial to produce in situ beyond LEO is aerogels. Aerogels, first created by Kistler in 1931, are human-made, low-density solid materials with an interconnected porous network composed of 99.8% air, with the most common type being silica-based^281^. Photosynthetic organisms that can produce silica, primarily algae diatoms (*e.g., Phaeodactylum tricornutum*), are of interest for the generation of silica-based aerogels and have recently been incorporated into cellulose aerogel composites^282,283^. Aerogels are useful materials for space applications due to their low thermal conductivity, light weight, and high porosity^284–286^. To improve the habitability of other planets for humans and photosynthetic organisms, silica aerogels provide the benefit of allowing for the transmission of visible light for photosynthesis while simultaneously blocking hazardous UV radiation^284^. In addition, since aerogels are a thermally insulating material, they can raise surface temperatures through the solid-state greenhouse effect^284^. NASA is currently taking advantage of the thermal insulating properties of aerogels to protect spacecraft and rovers from the cold Martian surface temperature, such as for the Mars Pathfinder lander, Mars exploration rovers (Spirit and Opportunity) and Mars Science Laboratory mission (Curiosity rover)^286^. Aerogels have also been used as a method for capturing particles from space without damaging them, as demonstrated in the Stardust Mission^286^. Therefore, using a synthetic biology approach to generate aerogels from silica-producing organisms could be interesting to investigate further.

Other gel-based materials can also be synthesized using synthetic biology. For instance, Kim et al. took advantage of the structural properties of synthetic spider silk^287,288^ and mussel adhesive proteins, which can act as biological adhesives^289,290^, to engineer *E. coli* with a spidroin-amyloid-mussel foot hybrid protein that can ultimately assemble into a hydrogel with high strength and underwater adhesion^291^. The hybrid protein consisted of a zipper domain from an amyloid protein, a flexible domain from spider silk, and a dihydroxyphenylalanine (DOPA)-containing mussel foot protein. This is a great example of how synthetic biology can be used to generate bacterial hosts expressing recombinant proteins with novel or desirable functions and properties, which can be applied to tackle specific manufacturing challenges in space. In addition, methods to spatially control the distribution of microbial cells into hydrogel structures have been developed using a Stereolithographic Apparatus for Microbial (SLAM) Bioprinting 3D printer^292^. Bioresins composed of synthetic polymers were used to contain the microorganisms and mimic extracellular polymeric substances (EPS) that are fundamental to biofilm formation in nature^293^. The power of this technology was demonstrated by printing engineered *Caulobacter crescentus* as uranium biosensors within this biomaterial using a uranium responsive promoter fused to GFP and measuring the fluorescence output^292^. This technology could enable genetic engineering of single strains, microbiomes, and biofilms to be used not only for biomanufacturing but also for biomining, biotherapeutics, and bioremediation.

#### Polymer production and degradation

Plastic materials continue to play a vital role in the manufacturing of spacesuits and spacecraft. Progress has been made towards the sustainable production of high-strength, biodegradable plastics in engineered microorganisms. Specifically, efforts have been made to increase the production of polyhydroxyalkanoate (PHA) or polyhydroxybutyrate (PHB) in engineered cyanobacteria, *Synechococcus*^294^ and *Synechocystis* sp. PCC6803^295,296^. *Synechocystis* sp. PCC6803 has been engineered with the PHA biosynthetic pathway of *R. eutropha* (i.e., Cupriavidus necator)^296^ or the overexpression of *sigE*^295^ to increase PHA and PHB production, respectively. Metabolic engineering of the shikimate pathway in *B. subtilis* or *S. cerevisiae* for the increased production of *para*-aminobenzoic acid (pABA) has also been studied, which can act as a precursor for high-strength polymers (e.g., aramid fibers)^297,298^. Ultimately, these biologically derived materials can be used to manufacture parts or even 3D-print hardware in space^299^.

To make in-space manufacturing and construction off-planet more sustainable, ISRU or recycling of existing material components to produce feedstock for new materials is necessary^300^. Microorganisms offer a solution to this problem as many can naturally degrade polymers for metabolic products. Black fungi, which are a diverse group of extremophilic melanized fungi, have been investigated for this application due to their demonstrated ability to hydrolyze synthetic polymers^301^). For instance, *Aureobasidium pullulans*, a black fungus, has demonstrated microbial deterioration of plasticized polyvinyl chloride (PVC) and dioctyl adipate plasticizers^302^. *Knufia chersonesos*, another black fungus, has been shown to completely break down the synthetic copolymer polybutylene adipate terephthalate (PBAT)^303^. Secretome screening identified seven polyesterase enzymes that could potentially be involved in this observed polymer degradation, which lays the foundation for the possibility of further engineering of this biosynthetic pathway for more efficient degradation^303^. Therefore, this group of extremophilic organisms are promising candidates for plastic degradation, however, this process still needs to be investigated in simulated or spaceflight microgravity.

Microgravity studies of *K. cheronesos* that analyzed the effects of simulated microgravity on the proteome and secretome found that scytalone dehydratase gene expression was upregulated in the wild-type strain and downregulated in a melanin-deficient mutant strain^304^. This enzyme is involved in the biosynthesis of dihydroxynaphthalene melanin, which is believed to have protective qualities that fungi use to withstand the extreme conditions of space^305^. This suggests that *Knufia* species, and potentially other black fungi have the properties to withstand space conditions and are good candidates for plastic degradation in space. Since then, various other extremophilic fungi have been tested for their survival in space, through exposure to simulated Mars conditions^306,307^. These include 12 Chernobyl-isolated strains (i.e., *Cladosporium*, *Acremonium*, *Beauveria*, *Fusarium*, *Trichoderma*, *Penicillium*, *Aureobasidium*, *Aspergillus* and *Apiospora*), the black fungi *Exophiala jeanselmei*, and the microcolonial fungi *Cryomyces antarcticus* and *Knufia perforans*. Plastic degradation has also been investigated in extremophilic bacteria, for example *Streptomyces thermoviolaceus*, *Geobacillus thermocatenulatus* and *Clostridium thermocellum*^308^. As these extremophilic fungi and bacteria have been shown to withstand space conditions, thus they are promising candidates to use or engineer for plastic degradation in space. Ultimately, the capacity for manufacturing in space will be beneficial for long-duration space missions, reducing the need to bring materials as cargo or have them launched from Earth, and allowing for on-demand production of materials based on immediate need.

### Myco-architecture

Astronauts venturing out beyond Earth’s protective magnetosphere will be exposed to hazardous radiation during deep-space exploration missions. This includes high-energy electromagnetic waves from our sun such as UV radiation, gamma, and X-rays or sub-atomic particles from the cosmos (electrons, protons, neutrons, and heavy metal ions), known as galactic cosmic radiation. These forms of radiation strip electrons from molecules resulting in protein or DNA damage through production of reactive oxygen and nitrogen species^309,310^. The result is short-term or long-term health problems such as cancer, acute radiation sickness, radiation-induced cardiovascular disease, and neurological damage^278^. Developing a solution for passive radiation protection for astronauts will be a critical step towards sustaining long-term presence on the Moon and Mars. The average person on Earth is exposed to about 6.2 mSv of radiation over a period of a year, while the average astronaut on the ISS is exposed to approximately 144 mSv^311^. One year into a three-year mission to Mars, an astronaut would already have been exposed to some 400 mSv of radiation^311,312^. Due to the complex nature of space radiation, there is likely no one-size-fits-all solution to this problem. Some proposed architectural concepts for radiation production include building below ground in lava tubes or piling meters of regolith outside of a structure^313,314^ and while materials like lead and aluminum may be effective, they would be costly to transport^315^. As a result, the search for innovative radiation shields will depend in part on biotechnology, which holds unique advantages such as suitability for ISRU, self-regeneration, and adaptability. By selecting model organisms such as extremophiles, that use radiation as an energy source, we can begin to understand their properties and refine testing for technology development.

Fungi on Earth have been isolated in high-radiation environments, such as the contamination zone of the Chernobyl Nuclear Power Plant^316,317^, inside the ISS^317^, and exteriors of spacecraft in LEO^305^. Analogous to phototrophy, fungi appear to perform radiosynthesis, using pigments known as melanin to convert gamma radiation into chemical energy^318,319^. Melanin has the capability to absorb electromagnetic radiation, resist acids, and perform powerful antioxidant activity allowing some fungi to thrive in the most extreme environments on Earth or beyond, including those with high levels of ionizing radiation^320,321^. Studies examining the survival rates of melanized and non-melanized yeasts with gamma radiation have shown that melanin-rich fungi were able to shield ionizing radiation at efficacies comparable to lead and twice as effective as charcoal, whereas the non-melanized strains lacked the capability to provide shielding^322^. This may not only be due to the presence of melanin itself but the spatial arrangement of it within the cell, as it was observed that in *Cryptococcus neoformans*, melanin arranged in a spherical shape, covering the inner surface of the cell membrane, resulted in superior shielding from radiation^323^, hypothesized to be due to the increase in scattering of incident photons^318^. Melanin is not the sole mechanism by which fungi survive radiation exposure. In a study using melanized yeast *Exophiala dermatitidis*, it was found that nutrient availability, culture density, metabolic state and DNA repair mechanisms were better determinants of cell survival after gamma radiation exposure than melanin^324^. Other mechanisms that protect fungi against radiation involve enzymes that remove reactive oxygen species or those that promote DNA repair, either through nucleotide excision or photoreactivation^325^, and various other secondary metabolites, such as the antioxidant pyranonigrin A^326^. Due to the ability of various fungi to withstand, thrive, and even attenuate space-relevant doses of radiation^306,316,318,327–330^ there is keen interest in their use for the development of radiation-resistant shields or structures^305,330–332^.

Fungal mycelium, a filamentous network of hyphae, is a fibrous material that can be used as structural components for the construction of habitats, buildings, furniture, etc.^333^. Fungal mycelium as a construction material has attractive characteristics including self-healing potential, impressive compressive strength, flexibility, insulation, and hydrophobicity^333^. The use of mycelium-based materials and structures in space would be a sustainable, biodegradable option with demonstrated uses for generating textiles^332^ and as an alternative to plastic packaging. Haneef et al. used two edible and medicinal fungi, *Ganoderma lucidum* and *Pleurotus ostreatus*, to produce mycelium films composed of polysaccharides, lipids, protein, and chitin^334^. The fungi were grown on two different substrates; cellulose or cellulose with PDB, and the final composition and characteristics of the film differed based on which substrate they were exposed to^334^. This suggests that myco-architecture properties could be modulated simply by varying the growth substrate. Biocomposites combining fungal mycelium with cellulose plant fibers^335^ or with wood and cellulose nanofibrils have also been investigated^336^. Since they are living organisms, synthetic biology could be used to engineer fungi to secrete other useful structural components such as polymers, for even more complex structures. Indeed, CRISPR-Cas9 methodology has been used to create gene deletions in *Paecilomyces variotii*, a Chernobyl fungal isolate, and was used to identify the gene responsible for its radiation resistance^337^. This technology could be further employed to enhance various fungi for deep space applications, such as making them better suited for radiation shielding.

### Concrete production

Another promising construction material to shield humans, plants and (possibly even) animals from the harsh Lunar and Martian environments is concrete. Concrete is a promising material for space applications as it is strong (it has proven to be the most durable material against natural disasters and extreme weather events), resistant to burns, rust and rot and could be made with engineered microbes and in situ resources. Concrete is composed of three main components: water, cement, and an aggregate (i.e., sand, gravel). Researchers have discovered that the use of human serum albumin combined with regolith from the Moon or Mars as the concrete aggregate can produce a concrete-like biocomposite that is made even stronger with the addition of urea^338^. Alternatives for cement, one of the main components of concrete, can be made using engineered bacteria to express recombinant or structural proteins, such as bovine or human serum albumin^339^ and spider silk^340^. This way, protein production and purification can be scaled-up and ultimately mixed with in situ regolith, rather than extracting them directly from the source.

Microbes can further aid in the production of concrete through soil biocementation using microbially induced calcite precipitation (MICP)^341^. This is a process wherein microorganisms precipitate calcium carbonate, the main component of limestone and cement, by urea hydrolysis or CO_2_-concentration^341^. For this reason, candidate microbes are typically highly urease-active bacteria, such as *Sporosarcina pasteurii* (formerly known as *Bacillus pasteurii*)^342,343^. One of the limitations of concrete is that it can often crack due to shrinkage. Incorporation of microorganisms into these structures can provide a sustainable solution for in-space construction but also a long-term biotechnological solution for concrete maintenance through crack remediation. Bacteria embedded in the concrete, resulting in what is often termed “living” or “self-healing” concrete, can repair cracks that form over time through the precipitation of calcium carbonate crystals^344^. This has been investigated using microorganisms including microalgae (*Synechococcus* and *Spirulina*)^345,346^, *Bacillus*^347^, and *Deinococcus radiodurans*^348^. Concrete is a harsh, alkaline environment (~pH 12) and therefore relies on hardy microbes tolerant of these conditions. The ability for *Bacillus* to form spores and its resistance to alkaline conditions makes this microbe a promising candidate for survival in the high pH concrete environment. Concrete remediation using MICP by spore-forming *Bacillus* species was demonstrated by Ramachandran et al.^349^ and later by Jonkers et al., who demonstrated that the spores remained viable for up to four months^350^. While this is a promising start, experiments need to be performed to determine whether bacterial spores can remain viable for longer durations. The *B. subtilis* gene cluster and molecular mechanism involved in calcium carbonate biomineralization have been identified^351^ and therefore could be used to engineer novel bacteria with enhanced self-healing capabilities or resistance to the concrete environment.

Using extremophilic bacteria that can tolerate both the concrete and space environment to produce calcium carbonate may allow for the construction of Lunar or Martian structures with increased durability or radiation resistance. One such candidate is the cyanobacterium *Synechococcus*, a photosynthetic organism that can withstand extreme environmental conditions. Using *Synechococcus* PCC8806, Zhu et al. incorporated cyanobacteria into a mixture of hydrated concrete powder and sand. Results showed that 38% more calcium was precipitated in conditions containing cyanobacteria than in controls, and the microbial calcite layer on the concrete surface was thicker, more adhesive, and decreased water absorption by twofold^345^. More recently, *Synechococcus* sp. PCC 7002, was tested for incorporation in a sand-hydrogel scaffold^352,353^. Following inoculation into a scaffold of sand, gelatin and media, the microbial viability was analyzed in varying temperature and humidity conditions. While the scaffold was strongest at ambient conditions, cells were not viable after seven days, however at 50% or 100% humidity, 9% and 14% of the cells remained viable after 30 days, respectively^352^. Since desiccation appeared to reduce cell viability, another study analyzed whether changes in component ratios or addition of trehalose would allow for increased viability. They found that using a desiccation protectant like trehalose led to increased cell viability at ambient temperature without sacrificing the mechanical properties of the structure^353^. Maintaining cell viability of cyanobacteria in the concrete structure is vital for enhancing the crack-healing potential, which was shown to be promising in a recent study using *S. elongatus* and *S. platensis* at varying concentrations (i.e., 4, 8 and 12%)^346^. An alternative strategy to combat desiccation would be the use of a desiccation-resistant organism such as *D. radiodurans*, which is a polyextremophilic bacteria known for its resistance to radiation. Unlike some of the other candidate bacteria, *D. radiodurans* is non-spore forming and therefore has applications for low-temperature conditions^348^. *D. radiodurans* was shown to be viable for up to 28 days in concrete, providing significant crack healing and increasing the compressive strength of the mortar by 42% at room temperature and 38% at near-freezing temperatures^348^. Strategies enlisting microbes in combination with Lunar or Martian regolith provide a blueprint for ISRU for manufacturing strong, radiation-resistant structures that could further space exploration efforts and the possibility for human habitation on the Moon and Mars.

## Negative impact of microbes and mitigation strategies

While microbes offer many benefits and biotechnological solutions for extended spaceflight and the development of sustainable habitats beyond LEO, they can also have a negative impact on space exploration. This can manifest as increased microbial pathogenicity, horizontal gene transfer (HGT) of problematic genes, and biofilm formation which have direct effects on crew health and spacecraft integrity. Understanding how microbes respond to the space environment, will allow for mitigation strategies to be developed and implemented, to ensure successful long-duration space exploration.

### Pathogenicity

Several studies have demonstrated that the conditions and stress of spaceflight can enhance microbial virulence or pathogenicity, as has been observed with many human-associated pathogens either grown or isolated on the ISS and/or in simulated microgravity conditions. Examples include *Klebsiella pneumonia*^354^, *P. aeruginosa*^355^, *Salmonella enterica* serovar Typhimurium^356,357^, *Serratia marcescens*^358^, enterotoxigenic *E. coli*^359^, and *Aspergillus fumigatus*^360^. The presence of microorganisms with heightened virulence could increase the risk of crewmembers contracting infections, even more problematic due dysregulated immunity during spaceflight^179^. *S. typhimurium* grown under modeled microgravity (MMG)^356^ and in spaceflight^357^ showed increased virulence compared to ground controls as demonstrated by an increased mortality rate in a murine infection model. Similarly, *S. marcescens*^358^ and the pathogenic fungi, *Aspergillus fumigatus*^360^, grown on the ISS or in simulated microgravity exhibited enhanced virulence compared to terrestrial strains and were more lethal in a *Drosophila* and zebrafish model, respectively. The specific mechanism responsible for increased microbial virulence in space conditions is still under investigation but genes with altered expression in-flight may contribute to this virulence response. These include biofilm-associated genes which were found to be upregulated in *S. typhimurium* (e.g., *wca/wza* genes for colanic acid synthesis, *ompA*, *fimH*) and bacterial motility genes which were downregulated^361^. In addition, transcriptional, and proteomic analysis of *P. aeruginosa* identified genes that were upregulated in spaceflight including virulence- and adhesin-associated lectin genes (*lecA* and *lecB*) and *rhlA* which is involved in biosurfactant production^355^. However, the most common factor contributing to space-induced virulence seems to be transcriptional regulation by Hfq, the expression of which was shown to be decreased under both low shear modeled microgravity (LSMMG) and spaceflight conditions in *S. typhimurium*^361,362^ and *P. aeruginosa*^355,363^. Hfq is an RNA chaperone and global transcriptional regulator responsible for controlling the expression of a large array of genes and is necessary for the virulence of several bacterial pathogens^355^. Consistent with previous studies, *hfq* was found to be downregulated in *S. aureus* grown in LSMMG, however, unlike previous studies the virulence potential was reduced^364^.

Conversely, some studies have concluded that while microorganisms with pathogenic potential are present in these space environments, spaceflight conditions do not lead to increased virulence or microbial characteristics that would directly impact crew health^365–367^. For example, using a macrophage infection assay, O’Rourke et al. found that there was no significant difference in the virulence of *Burkholderia* species isolated from the ISS compared to Earth controls^368^. In addition, investigation of four common clinical pathogens, *Listeria monocytogenes*, methicillin-resistant *S. aureus*, *Enterococcus faecalis*, and *C. albicans* revealed that they were less virulent in space regarding their ability to kill *Caenorhabditis elegans* nematodes^369^. Similar conclusions were drawn in the ISS experiment EXTREMOPHILES, where sequencing analysis and physiological tests were performed on microbial communities from several surfaces aboard the spacecraft^370^. Researchers proposed that while the ISS environment selects for bacteria with more natural resistance and tolerance to extreme conditions, it does not induce genetic or phenotypic changes that result in more extremophilic, or antibiotic resistant bacteria compared to a built, enclosed environment on Earth^370^. Indeed, a pan-genomic analysis of microbes from the ISS and built environments on Earth found that the antimicrobial resistance (AMR) genes present in ISS samples were also present in control samples, and that functional changes common to built environments do not have a direct impact on astronaut health^371^. Collectively, these studies demonstrate the impact of spaceflight on individual pathogenic microbes in built environments and, for many, it remains to be determined if the virulence phenotype depicted in their models will directly translate to mixed bacterial populations or lead to enhanced pathogenicity in humans. Therefore, assessing the microbiome within the host and continuing to elucidate the mechanism involved in potential spaceflight-enhanced virulence will be necessary. Although there is some debate regarding the increased pathogenicity of microbial populations in space, there is evidence of indirect health concerns caused by harmful biofilm formation^370^.

### Biofilms

Microorganisms in the space environment, as on Earth, can exist in a planktonic (freely suspended) state, but more commonly form robust biofilms as a tactic for growth and survival. A biofilm is an assemblage of surface-associated microbial cells surrounded by an extracellular matrix of polysaccharides, extracellular DNA (eDNA), proteins, lipids, and other components, with a defined architecture^372,373^. Biofilm-associated organisms differ from their planktonic counterparts with respect to the genes that are transcribed, proteins that are translated and growth rate^372^. Although most bacterial biofilms are harmless, some threaten human health and safety and can be difficult to eradicate due to increased resistance to the immune system’s defenses, UV radiation, extreme temperatures, pH, high salinity, high pressure, limited nutrients, and various antimicrobials^374,375^.

Environmental stressors are known to induce biofilm formation^374^ and spaceflight is one such stressor. The impact of microgravity on biofilm formation was investigated for the first-time using *P. aeruginosa*^376^, and later during two NASA-funded studies, Micro-2 and Micro-2A^377^. In these NASA studies, P. *aeruginosa* grown on the ISS displayed different biofilm characteristics compared to ground controls such as an increased number of viable cells, biomass, and thickness and a novel column-and-canopy shaped architecture^377^. However, this novel architecture was only observed in biofilms formed by motile bacteria, as non-motile strains produced flat structures similar to those seen with the ground controls^377^. In another early biofilm investigation experiment conducted in space, *Burkholderia cepacia was* grown in sterile water, tryptic soy broth (TSB), and an iodine solution (a disinfectant), for six days on stainless-steel coupons^378^. Results showed that bacteria grown in space, in sterile water, had a biofilm plate count (measured as CFU/m^2^) five times larger compared to ground controls, however, the space grown TSB population was one quarter of that on Earth^378^. Those grown in the iodine solution in space, also had a higher biofilm growth compared to the ground controls, suggesting increased resistance to disinfectants during spaceflight^378^. In addition, spaceflight conditions have been demonstrated to upregulate gene expression for the production of extracellular matrix proteins leading to enhanced cell aggregation compared to ground controls in *C. albicans*^361^. Differential expression of genes related to motility, which is important for the formation of biofilms, has also been observed in flight conditions^379^.

#### Biofilms and biodeterioration

Microbially influenced corrosion (MIC), refers to the deterioration of metals and nonmetallic materials due to microbial activity, most often due to biofilms. As biofilms increase in quantity more microbial-surface reactions occur, enhancing structural and/or functional damage, causing accelerated biocorrosion^380^.

In a recent Microbial Tracking study (MT-1) of the ISS, the bacterial bioburden quantified from various surfaces was as high as 10^9^ CFU/m^2^^381^. Some of the biofilm-forming microorganisms that were identified, *Methylobacterium, Sphingomonas, Bacillus, Penicillium*, and *Aspergillus* have been implicated in MIC on Earth^382–385^ with *Bacillus polymira*, *Penicillium rubens* and *Aspergillus* sp. responsible for progressive destruction of a navigation window on board Mir^386^. *Sphingomonas sp*. and *Methylobacterium sp*. have not only been detected on surfaces but also in portable drinking water on the ISS^387^. Over the course of 15 years (from its launch in 1986 to 2001), 234 species of bacteria and fungi were identified onboard the MIR space station, with many exhibiting potential polymer biodegradation properties^388^.

Biofilm growth has been observed in the Soviet/Russian (Salyuts and Mir), American (Skylab), and International (ISS) Space Stations. Aboard spacecraft, biofilms can jeopardize vital equipment and threaten astronaut health by corroding surfaces or clogging life-support systems including air and water purification systems, spacesuits, navigation windows and radiators^368,389–392^. Most notable on the ISS, is the microbial contamination and biofilm formation that occurs in the wastewater tank of the Water Recovery System (WRS), which is a part of the Environmental Control and Life Support System (ECLSS) and used to process wastewater from various sources (i.e., urine, cabin condensate) into potable water for crew and other functions^393^. For future planned missions beyond LEO to the Moon and Mars, resupplying spare parts or support materials to repair the listed spaceflight systems would be impractical as missions could be in the order of years, in the case of a Mars missions, thus various strategies to control biofilms, especially in critical life support systems are essential^394^. Ways to detect, monitor and control biofilms are being explored, such as the current spaceflight BAC (Bacterial Adhesion and Corrosion) study. The aim of this study is to identify bacterial genes relevant to biofilm growth in space, examine whether the formed biofilms corrode stainless steel surfaces mimicking those in the ISS water system, and determine whether silver-based disinfectants can prevent or control extensive biofilm formation.

#### Biofilms and astronaut health

Biofilm formation is an important characteristic in the infectious disease process of microorganisms. It has been demonstrated that bacteria can genetically and physically modify their tolerances to LEO conditions, with one such mechanism being biofilm formation^395–397^. Human opportunistic pathogens that form or increase biofilms under simulated microgravity conditions or when grown on the ISS include *E. coli*, *S. typhimurium*, P. *aeruginosa* and *Micrococcus luteus*^379,398^. It has also been observed that many species of bacteria and fungi become more antibiotic resistant and pathogenic when exposed or grown in spaceflight conditions^174^. In cases of *S. typhimurium*^17^ and *P. aeruginosa*^355^, the observed increased virulence (discussed earlier in this review) was attributed to molecular and phenotypic changes consistent with biofilm formation. In the case of *Klebsiella pneumoniae* grown aboard the Shenzhou VIII spacecraft, the enhanced antibiotic resistance was associated with adaptations related to biofilm formation^354,399^. As biofilm formation can increase the risk of human illnesses, through harder-to-treat infections, biofilm properties under space conditions need to be well understood to enable safe, long-duration, human space missions. This is even more imperative considering the immune dysregulation of astronauts and lower efficacy of pharmaceuticals during spaceflight^174^.

#### Positive impact of biofilms

While biofilms can be detrimental to astronaut health and structural stability, they can be beneficial in areas such as plant protection, bioremediation, wastewater treatment, and corrosion inhibition, amongst others^400^. Thus, strategies for manipulating biofilms should not focus on complete eradication, but rather regulation, to promote the growth of beneficial ones while inhibiting the growth of harmful ones^400^. These beneficial applications of biofilms may also be extended to spaceflight. Ichikawa et al. developed a long-term life support system that uses an electrochemically activated biofilm reactor^401^. This system was tested for the removal of nitrate produced from biological nitrification and is an important process to allow for long-term survival of aquatic organisms in a closed system^401^. Results from this biofilm-electrode reactor study showed that neither ammonia nor nitrite accumulated, and nitrate could be suppressed to about 10 ppm^401^. Biofilms can also provide insight into how humans tolerate spaceflight. Biological dosimetry is an internationally approved method to perform an exposure assessment following a suspected radiation overexposure. In contrast to physical methods, which measure the actual dose, biological dosimeters measure dose effects, at the cellular level, when assessing the impact of radiation exposure on humans^402^. In the BIODOS project, four DNA-based biological dosimeters (phage T7, uracil thin layer, spores, and biofilms) were validated for their effectiveness in determining the biological hazards of environmental UV exposure (i.e., sunlight), and were shown to be reliable field dosimeters^403^. This same biofilm dosimeter was then used in a study by Rettberg et al. to determine the biological effectiveness of the UV radiation climate at different locations in the space station, with the aim of ensuring that astronauts had enough UVB to synthesize vitamin D^404^. Conclusions from this study showed that the amount of UV radiation inside the station was not sufficient for an adequate supply of vitamin D and that specialized UV lamps were needed to maintain healthy levels of Vitamin D for astronaut health^404^. Overall, more work on biofilm applications for spaceflight is needed to tease out the benefits biofilms may offer, while reducing any harmful properties.

### Horizontal gene transfer

The ability for bacteria to survive or even thrive in the spaceflight environment, with potentially increased pathogenicity and biofilm formation, may be attributed to DNA transfer. Horizontal gene transfer (HGT), also known as lateral gene transfer, is the movement of genetic material from one organism to another by means other than sexual reproduction or vertical transfer from a parent cell. This typically occurs through one of three main mechanisms: direct contact of microbial cells by a pilus (i.e., conjugation), natural DNA uptake from the environment (i.e., transformation), or introduction by bacteriophages (i.e., transduction)^405–407^. More recently, gene transfer agents and membrane vesicles are being recognized for their contributions to HGT as well^408^. HGT is central to microbial evolution because it allows microorganisms to acquire novel genetic material, which may confer a fitness advantage to adapt to or thrive within a specific environment^22,409–411^.

#### Prevalence of HGT in the spaceflight environment

In the sealed spaceflight environment, bacteria must adapt to extreme conditions including microgravity and cosmic radiation^412^, which could have an impact on the prevalence of genetic transfer. The three main mechanisms of HGT (transduction, transformation, and conjugation) were first investigated for their occurrence in space by Ciferri et al. during the Spacelab D1 mission (STS-61-A, 1986)^413^. They discovered no significant difference in transduction, inconclusive transformation results, and increased transmission of antibiotic resistance genes by conjugation in *E. coli* exposed to microgravity^413^. It was postulated that this increase in conjugation frequency could be due to a decrease in mating pair disruptions in microgravity compared to Earth gravity, as continuous cell-to-cell contact is required for conjugation to occur. Another spaceflight experiment was performed on the Discovery Mission (STS-63) where researchers concluded that transformation efficiency to *E. coli* was decreased in microgravity^414^. Later, during the Soyuz Mission 8S on the ISS, the Mobilisatsia/Plasmida experiment examined plasmid-mediated conjugation through triparental mating of both Gram-positive and Gram-negative bacteria^415^. They found that conjugation was increased in experiments between *Bacillus thuringiensis* strains (Gram-positive), while no trends were observed in conjugation experiments from *E. coli* to *C. metallidurans* (Gram-negative)^415^. This increase in conjugation efficiency could be attributed to the mechanistic differences between Gram-positive and Gram-negative conjugation, as Gram-positive bacteria facilitate contact between donor and recipient cells through surface adhesins rather than conjugative pili^416^. However, authors caution the drawing of conclusions due to failure to obtain transconjugants in some replicates of ground controls. Conversely, conjugation experiments performed using the same Gram-positive species (*B. thuringiensis*) in simulated microgravity showed that there was no significant difference in plasmid transfer frequency for a plasmid mobilizing itself (*cis*) or mobilizing a separate plasmid (*trans*) compared to standard laboratory conditions^417^. Most recently, Urbaniak et al. tracked HGT of two antimicrobial resistance (AMR) genes by co-culturing of two species, *Acinetobacter pittii* and *S. aureus*, isolated from the ISS as part of the Microbial-1 tracking study^381^. The results indicated an approximately 100-fold increase of HGT in simulated microgravity compared to Earth gravity controls (1-*g*)^418^. Combined, these results indicate that HGT occurs in spaceflight conditions within and between Gram-negative and Gram-positive bacteria and is typically not hampered, and is more often increased, by spaceflight conditions.

#### HGT and astronaut health

The human gut houses a diverse microbial population with ecologically favorable conditions for HGT given its continuous supply of nutrients and consistent environmental conditions (e.g., temperature, pH)^14^. Phylogeny-based research suggests that over the course of their evolution, more than half of total genes in the genomes of human-associated microbiota were introduced by HGT^419^. While HGT is a normal occurrence within the human microbiome, an increased number of transfer events passing AMR genes from commensal bacteria to opportunistic pathogens during spaceflight could cause changes in these microbial communities^175^. These transfer events have been investigated in *Staphylococcus epidermidis*, a bacterium normally present in the human epithelial microbiome, which harbors the methicillin resistance gene *mecA* on a mobile genetic element. HGT was found to be an important factor for the acquisition of *mecA* by the pathogen *S. aureus*, leading to methicillin-resistant *S. aureus* (MRSA)^420,421^. Recent research has also demonstrated the transfer of AMR and virulence-associated genes from commensal *E. coli* to pathogenic *E. coli*^422^. The transfer of AMR genes can cause increased bacterial resistance and virulence, which could have significant health implications^423,424^ for astronauts due to their dysregulated immunity on long-duration space missions^425^. Numerous studies have shown that HGT is the common mechanism by which AMR genes are disseminated within an environment, leading to the emergence of multi-drug resistant bacteria^426–428^, which limits treatment options for bacterial infections.

Some research has proposed that the increased virulence or pathogenicity observed in some organisms grown under spaceflight conditions is attributed to possible increased HGT in spaceflight conditions^357,429,430^. In a study comparing similar species from two extreme built environments, one on Earth (Concordia Research Station in Antarctica) and one in space (ISS), it was observed that 76% of the isolates from the ISS were resistant to one or more antibiotics tested compared to only 44% of the Concordia isolates^429^. This increased resistance amongst the ISS isolates could be attributed to the higher number of mobile genetic elements (involved in HGT) within their genomes compared to the Antarctic strains^429^. The reason for this increase in mobile genetic elements is unclear but it could be a response to the unique stressors of the space environment. Further, in a study published by Urbaniak et al. examining the ISS ‘resistome’, whole genome sequencing revealed AMR gene clusters in *Enterobacter bugandensis* isolated from the waste and hygiene compartment on the ISS. Further examination showed that these isolates shared AMR gene clusters with known pathogens from different genera which were not present in any *Enterobacter* species isolated on Earth^430^. The researchers proposed that these AMR genes may have been acquired through HGT, but further analysis is necessary to conclude this with certainty. Changes in HGT-associated gene expression were also identified in ISS-derived isolates of *S. typhimurium* during Space Shuttle mission STS-115, which exhibited enhanced virulence and increased biofilm formation in a mouse model compared to the ground control^357^. The expression of *hfq*, an RNA chaperone and negative regulator of the F plasmid-encoded *tra* genes, was decreased in spaceflight. *TraJ* is an activator of the *tra* operon, and *hfq* has been shown to specifically repress *traJ* expression by destabilizing its mRNA^431^. As a result, several *tra* genes were upregulated in response to spaceflight^357^. Mating assays performed in an *hfq* mutant showed an increase in protein levels of TraJ confirmed by immunoblotting as well as increased conjugation efficiency compared to wild-type controls^431^. Due to the multiple AMR genes found on the environmental surfaces of the ISS^430^ and possible enhanced HGT activity during spaceflight, further studies into HGT and the effect on crew health are is important to understand for the development of mitigation strategies^432–434^. In addition, further elucidation of differentially expressed genes and their effect on HGT could identify novel gene targets for modulating or reducing HGT in the space environment.

#### HGT and biofilms

As previously discussed, biofilms can be detrimental to spacecraft and astronaut health; thus, understanding the factors that can influence biofilm formation will be imperative for predicting, preventing, and mitigating spacecraft contamination. HGT may contribute to biofilms as their formation can be induced by conjugative plasmids that express factors to enhance cell-to-cell contact and pilus formation. Conjugative pili can act as adhesion factors as demonstrated by Ghigo who monitored biofilm formation on Pyrex slides submerged in cultures of *E. coli* K12. They observed that strains carrying a conjugative F plasmid (F+) formed thick biofilms after one day (2 × 10^10^ CFU/cm^2^), while plasmid-free strains (F−) only formed microcolonies (8 × 10^5^ CFU/cm^2^)^435^. Conjugative pili specifically were shown to be a contributing factor to biofilm formation as strains carrying plasmids with mutations in the pilin gene, *traA*, were unable to form biofilms^435^. Reisner et al. confirmed Ghigo’s findings as the presence of the F plasmid pOX38 in *E. coli* led to the formation of mushroom-shaped biofilms with increased biomass, surface coverage and thickness compared to F- controls^436^. To further analyze the contribution of the pilus to this phenotype, the authors created plasmid mutants for several of the genes required for pilus synthesis: *traQ*, *traX*, *traD*, *traS*, and *traT*^436^. Mutants involved in pilus assembly and modification (i.e., *traQ, traX*) displayed a weak biofilm phenotype similar to the plasmid-free strain (*traQ*) or decreased biomass and thickness (*traX*), while those involved in DNA transfer (i.e., *traD*) displayed rapid confluent growth and tower-like structures^436^. In a different study performed with 403 natural *E. coli* isolates researchers observed biofilm formation in 56 isolates and of those, 89% contained conjugative plasmids^437^. These recipients of conjugative plasmids were able to induce biofilms to a greater extent than their plasmid-free controls^437^. Finally, one study validated that conjugative plasmids enhanced biofilm formation but concluded that this did not directly correlate with conjugation frequency for all plasmids tested, suggesting other factors may be involved^438^.

Other studies suggest that conjugative plasmid-encoded fimbriae, biofilm-associated pili, and cell wall-anchoring proteins can also increase biofilm formation. Type III fimbriae encoded as accessory proteins on conjugative plasmids have been demonstrated to mediate cell–cell and cell-surface adhesion^439^, as well as increased biofilm formation^440^ in *K. pneumoniae*^441^. Further evidence of this was demonstrated in a uropathogenic strain of *E. coli* where Tn5 mutagenesis was used to identify biofilm-deficient mutants^442^. All Tn5 insertions were found to be within the type III fimbriae genes (*mrkABCDF*) encoded on the conjugative plasmid pMAS2027. This suggests that type III fimbriae were necessary for biofilm formation which was confirmed when this locus was cloned into biofilm-deficient mutants and was able to restore biofilm formation^442^.

When analyzed in a space environment it appears that pili and fimbriae contribute to increased biofilm formation in the short term but decreased biofilm formation in the long term. In a spaceflight study, researchers compared biofilm formation of *Acinetobacter schindleri* over a short duration (15 days) and long-duration (64 days) from the Shenzhou-10 spacecraft and Tiangong-2 space lab, respectively^443^. They observed reduced biofilm formation after 64 days and following transcriptional analysis proposed that this reduction was potentially due to downregulation of the *pil* and *algR* genes associated with conjugative pili and alginate biosynthesis, or upregulation of genes involved in metal iron binding (as available iron increases biofilm formation)^443^. In another study analyzing *Proteus mirabilis* biofilm formation following short-term or long-term SMG, researchers found that long-term exposure resulted in downregulation of genes associated with fimbriae, impeding adhesion, and ultimately decreasing biofilm formation^444^.

**Fig. 4:**
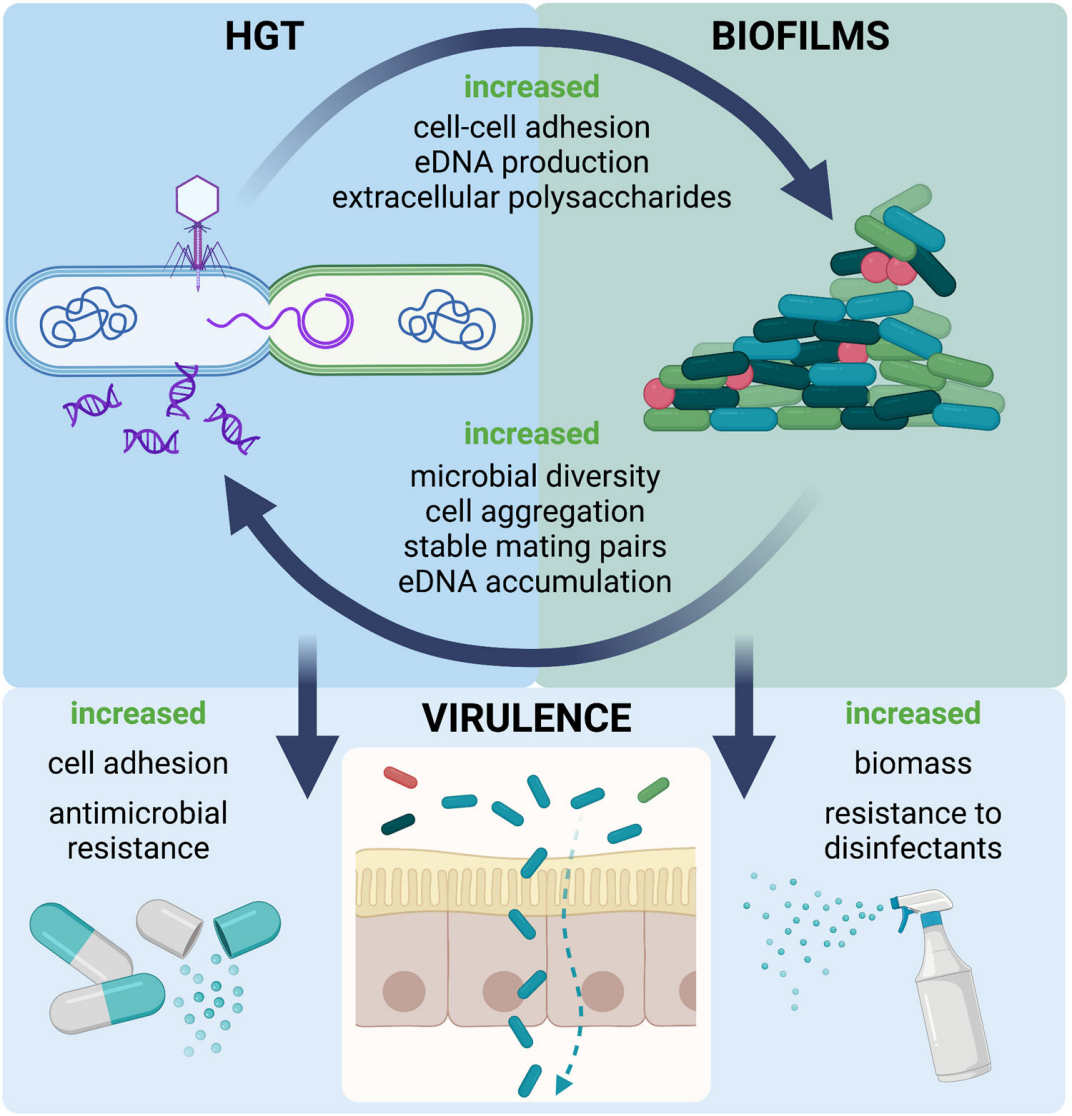
Interplay between bacterial HGT, biofilms, and virulence in space. In microgravity, bacterial HGT can increase cell–cell adhesion, and production of eDNA and extracellular polysaccharides contributing to increased biofilm formation. Reciprocally, biofilms promote HGT as they contain high microbial diversity and an ECM structure allowing for increased cell aggregation, stable mating pair formation and eDNA accumulation. Both HGT and biofilms increase bacterial virulence through cellular adhesion and dissemination of antimicrobial resistance, and increased biomass and resistance to disinfectants, respectively. Created with BioRender.com.

The rate of HGT amongst bacterial communities in biofilms is increased as it contains a diversity of bacteria, the structured extracellular matrix (ECM) provides the ideal environment to stabilize mating pair formation and bacterial contact for genetic exchange, and eDNA accumulates within the ECM allowing for natural transformation^435,445–447^. Lécuyer et al. analyzed the conjugative transfer of an integrative and conjugative element (ICE) between *B. subtilis* strains on normal media compared to biofilm-inducing media. Results indicated that conjugative transfer was increased by 100-fold and 10,000-fold on biofilm-inducing media using minimal media and rich media, respectively^446^. Conjugation experiments performed between *S. aureus* strains using donors harboring a mobilizable plasmid and the multi-drug resistant conjugative plasmid, pGO1 yielded similar results^448^. Conjugation was performed with standard filter mating or a cellulose disk static biofilm model and conjugation frequency in the biofilm-promoting conditions was found to be ~16,000-fold higher than in standard conditions^448^. Overall, HGT promotes biofilm formation which, in turn, promotes HGT transfer, and both can contribute to increased bacterial pathogenicity (Fig. 4). If HGT is increased under space conditions which can consequently lead to the production of more biofilms, the effect that biofilms have on spacecraft integrity and function could be even more severe during long-duration space travel if this feedback loop goes uninterrupted.

#### Harnessing HGT as a tool for genetic engineering

Although HGT has the potential to negatively impact astronaut health and jeopardize spacecraft during long-duration space missions, conjugation machinery can also be harnessed as a tool for the development of novel mitigation strategies. Conjugation has been demonstrated within and between many species of Gram-negative and Gram-positive bacteria in a laboratory setting, and from bacteria to eukaryotic cells. Conjugative tool development for many of these species could have implications for their use in the space environment including those developed to target opportunistic pathogens (e.g., *Enterococcus*, *Listeria, Staphylococcus*^449^*, Streptomyces*^450^, and *S. enterica*^451^), or those that could be used to engineer useful strains such as probiotic candidates (e.g., *Bacillus*^449^, *Bifidobacterium*^452^ and *Lactobacillus*^453^) or extremophilic microbes (e.g., *D. radiodurans*^454^, yeast^455^ or algae^267,268^). When coupled with CRISPR/Cas technologies conjugation can be used for the modulation of microbiomes and as a method for targeted eradication of human pathogens on Earth. This has been demonstrated using a *cis*- and *trans*- conjugative plasmid encoding the I-TevI nuclease domain fused to *Streptococcus pyogenes* or *S. aureus* Cas9 (TevSpCas9 or TevSaCas9) targeting *S. enterica*^451^. This study demonstrated *S. enterica* killing efficiencies ranging from 1 to 100% following conjugation using 65 single or multiplexed sgRNAs, allowing for the ability to modulate bacterial populations based on the chosen sgRNA. Another study using a similar strategy and a *trans*-conjugative system has demonstrated specific bacterial killing and targeting of a plasmid-born carbapenem resistance gene in *E. coli*^456^. Other Cas nucleases can be used to target pathogens as well, including CRISPR/Cas13a-based antibacterial nucleocapsids, which have been shown to kill resistant *E. coli* and *S. aureus* species by targeting AMR genes^457^. CRISPR/Cas technologies can also be used to inhibit conjugation in human pathogens^458,459^, including species that have been isolated from the ISS such as *K. pneumonia*^460^, *E. faecalis*^461^, and *Staphylococcus*^462^. The biotechnological applications of CRISPR/Cas systems in space are extensive, from using Cas proteins to understand the biological impact of microgravity to detecting the presence of pathogenic bacteria or viruses, as demonstrated on Earth using Cas12 or Cas13 for SARS-CoV-2 RNA detection^463–465^. The use of CRISPR/Cas systems for these in-space applications is feasible as genome editing using a CRISPR-based assay has been performed in *S. cerevisiae* onboard the ISS^466^. The mitigation strategies discussed here will require further testing and adaptation for space which could be carried out in synthetic human gut microbiomes^467^ or in simulated spaceflight biofilms^394^. However, these results indicate that harnessing the power of conjugation and CRISPR/Cas technologies could provide a promising strategy for detection of pathogenic bacteria, mitigation of HGT and modulation of human and environmental microbiomes in spacecraft.

### Mitigation strategies

To minimize the health risks associated with extended spaceflight, it is critical that methods for preventing and treating spaceflight-induced illnesses be developed before astronauts embark upon long-duration space missions. Specifically, increased HGT, pathogenicity and biofilm formation by microorganisms in the space environment presents the need for highly effective antimicrobials and targeted solutions for use on spacecraft. Aside from traditional antibiotics^468^, the use of live engineered organisms, and the use of the conjugation and CRISPR-based strategies discussed above, other mitigation strategies have been investigated to inhibit HGT and the development of biofilms^394^. One well-studied method is antibacterial photodynamic inactivation employing the use of a photoactive compound which accumulates in target bacteria where it is illuminated to produce a cytotoxic reaction with oxygen resulting in cellular damage and bacterial death (reviewed in ref. ^469^). Additional examples of mitigation strategies include the use of specialized material coatings or surfaces (e.g., copper-based antimicrobial surfaces^470,471^, silver and ruthenium bio-deterrent surfaces^472^), engineered lantibiotics^473^, chemical water treatment, UV light, sound waves, and phenols^474^. Other promising antimicrobial materials include AGXX^472^, which was shown to prevent the growth of *Staphylococcus* and *Enterococcus* pathogens on the ISS, and sterilization using cold atmospheric plasma^441^. Some potential antibiofilm coating issues that need to be explored include the longevity and chemical stability of the coatings^475^. While several coatings give extremely promising results in the short term, their chemical and physical stability may diminish over time^476^. Regarding long-term space exploration, replenishing these antimicrobial coatings will prove challenging. The use of plant-based extracts is thus gaining in popularity for their antimicrobial and antibiofilm properties along with the added benefit of being able to harvest the necessary compounds during spaceflight, as needed^390^.

## Future outlook and summary

Within the next decade, great strides will be made in space exploration with the combined efforts of NASA and other space agencies to reach the Moon and Mars, as well as the expansion of commercialized spaceflight. The Artemis missions will work towards establishing a lunar base camp by 2025 with the purpose of utilizing the Moon as a precursor to Mars exploration. These missions will involve long-duration spaceflight and prolonged extraterrestrial occupation, requiring further exploration of health risks and resource sustainability than what was needed for the decades spent in LEO on the ISS. This review has discussed the role of microbes and their beneficial or detrimental impacts on long-duration space missions as demonstrated in Earth-based simulated space environments or experiments conducted in spaceflight. Microbes heavily contribute to the success of our ecosystem on Earth and can therefore be repurposed for the space environment to help generate a sustainable habitat. We have summarized the advantageous properties of microbes in plant cultivation, wastewater treatment, solid and liquid waste reuse, and atmospheric revitalization. This review has also considered the use of microbial fuel cells to generate energy from waste, plant decay, and solar light conversion. Although more research is needed for efficient energy conversion in the space environment, we present microbial fuel cells as an ideal and possible future alternative for energy generation. We also examined the advantages of bioengineered microbes for in-flight therapeutics, increased production of nutrients and oxygen, and the creation or recycling of infrastructure materials. Most of these microbial technologies have been successfully demonstrated on Earth, but as suggested, should be further tested, and applied in the space environment. Microbes are ideal candidates to aid in human survival during long-term space travel due to their many beneficial characteristics, ease of manipulation, and microscopic size, allowing them to be easily stowed without the concern of added weight. In terms of health and safety risks, the possibility of increased microbial virulence and pathogenicity in spaceflight was discussed, as well as the detrimental effects of increased HGT and biofilm formation on astronaut health and equipment integrity. Suggestions were also made regarding mitigation strategies for further investigation, including antimicrobial metallic surface materials and coatings that prevent increased HGT or biofilm formation. This review has presented the vast contributions that microbes can make towards future crewed missions, human habitability and sustainability and hopefully encourages more space-related microbial research interconnected with technological development, to continue to safely advance deep space exploration in the upcoming decade.

## References

[1] Loff, S. & Lind, R. Beyond Earth Expanding Human Presence into the Solar System. https://www.nasa.gov/exploration/home/why_moon.html (2011).

[2] Dunbar, B. Artemis. https://www.nasa.gov/what-is-artemis (2021).

[3] Artemis Plan NASA’s Lunar Exploration Program Overview. https://www.nasa.gov/sites/default/files/atoms/files/artemis_plan-20200921.pdf (2020).

[4] How Investing in the Moon Prepares NASA for First Human Mission to Mars. https://www.nasa.gov/sites/default/files/atoms/files/moon-investments-prepare-us-for-mars.pdf (2021).

[5] *Orion Reference Guide*. (NASA Johnson Space Center). https://www.nasa.gov/sites/default/files/atoms/files/orion_reference_guide_0.pdf (2022).

[6] Mars, K. Gateway. NASA. https://www.nasa.gov/gateway/overview (2023).

[7] Atkinson, J. & Spears, S. NASA Seeking BIG Ideas for Solar Power on Mars. https://www.nasa.gov/press-release/langley/nasa-seeking-big-ideas-for-solar-power-on-mars (2017).

[8] Mars Report: Dust Storms on Mars. https://mars.nasa.gov/resources/26555/mars-report-dust-storms-on-mars/?site=insight (2022).

[9] Kawamoto H, Uchiyama M, Cooper BL, McKay DS (2011). Mitigation of lunar dust on solar panels and optical elements utilizing electrostatic traveling-wave. J. Electrostat..

[10] Stubbs TJ (2011). On the role of dust in the lunar ionosphere. Planet Space Sci..

[11] NASA Mars 2020 Mission Perseverance Rover Cruise. https://mars.nasa.gov/mars2020/timeline/cruise/.

[12] NASA Mars 2020 Mission Perseverance Rover Communications. https://mars.nasa.gov/mars2020/spacecraft/rover/communications/.

[13] Gilbert JA (2018). Current understanding of the human microbiome. Nat. Med..

[14] Cho I, Blaser MJ (2012). The human microbiome: at the interface of health and disease. Nat. Rev. Genet..

[15] Pickard JM, Zeng MY, Caruso R, Núñez G (2017). Gut microbiota: role in pathogen colonization, immune responses, and inflammatory disease. Immunol. Rev..

[16] LeBlanc JG (2013). Bacteria as vitamin suppliers to their host: a gut microbiota perspective. Curr. Opin. Biotechnol..

[17] Batista BD, Singh BK (2021). Realities and hopes in the application of microbial tools in agriculture. Micro. Biotechnol..

[18] Abhilash PC, Dubey RK, Tripathi V, Gupta VK, Singh HB (2016). Plant growth-promoting microorganisms for environmental sustainability. Trends Biotechnol..

[19] dos Santos Lopes, M. J., Dias-Filho, M. B. & Gurgel, E. S. C. Successful plant growth-promoting microbes: inoculation methods and abiotic factors. *Front. Sustain. Food Syst.* **5**, (2021).

[20] Caplice E (1999). Food fermentations: role of microorganisms in food production and preservation. Int. J. Food Microbiol..

[21] Thompson LR (2017). A communal catalogue reveals Earth’s multiscale microbial diversity. Nature.

[22] Webster NS, Reusch TBH (2017). Microbial contributions to the persistence of coral reefs. ISME J..

[23] Gougoulias C, Clark JM, Shaw LJ (2014). The role of soil microbes in the global carbon cycle: tracking the below-ground microbial processing of plant-derived carbon for manipulating carbon dynamics in agricultural systems. J. Sci. Food Agric..

[24] van der Heijden MGA, Bardgett RD, van Straalen NM (2008). The unseen majority: soil microbes as drivers of plant diversity and productivity in terrestrial ecosystems. Ecol. Lett..

[25] Barberán A (2014). The microbial contribution to macroecology. Front. Microbiol..

[26] Horve PF (2019). Building upon current knowledge and techniques of indoor microbiology to construct the next era of theory into microorganisms, health, and the built environment. J. Exposure Sci. Amp. Environ. Epidemiol..

[27] Lax S (2017). Bacterial colonization and succession in a newly opened hospital. Sci. Transl. Med..

[28] Drake, B. G., Hoffman, S. J. & Beaty, D. W. Human exploration of Mars, Design Reference Architecture 5.0. in *2010 IEEE Aerospace Conference* (IEEE, 2010). 10.1109/aero.2010.5446736.

[29] Microbiologist, A. J. The BioHome: A spinoff of space technology. *NASA, Washington, Biological Life Support Technologies: Commercial Opportunities* (1990).

[30] Salisbury FB, Gitelson JI, Lisovsky GM (1997). Bios-3: Siberian experiments in bioregenerative life support. Bioscience.

[31] Walker J, Granjou C (2017). MELiSSA the minimal biosphere: human life, waste and refuge in deep space. Futures.

[32] Fu Y (2016). How to establish a bioregenerative life support system for long-term crewed missions to the moon or mars. Astrobiology.

[33] Arena C (2019). Suitability of Solanum lycopersicum L. ‘Microtom’ for growth in Bioregenerative Life Support Systems: exploring the effect of high-scpLET/scpionising radiation on photosynthesis, leaf structure and fruit traits. Plant Biol..

[34] Desiderio A (2019). Effects of simulated space radiations on the tomato root proteome. Front. Plant Sci..

[35] Zhang J (2020). Competitive growth assay of mutagenized Chlamydomonas reinhardtii compatible with the international space station veggie plant growth chamber. Front. Plant Sci..

[36] Barker R, Lombardino J, Rasmussen K, Gilroy S (2020). Test of Arabidopsis space transcriptome: a discovery environment to explore multiple plant biology spaceflight experiments. Front. Plant Sci..

[37] Heiney, A. Growing Plants in Space. (2019).

[38] Massa GD (2017). VEG-01: Veggie hardware validation testing on the international space station. Open Agric..

[39] Hummerick ME (2021). Spatial characterization of microbial communities on multi-species leafy greens grown simultaneously in the vegetable production systems on the international space station. Life.

[40] Jarvis WR (1989). Managing diseases is greenhouse crops. Plant Dis..

[41] Bishop DL, Levine HG, Kropp BR, Anderson AJ (1997). Seedborne fungal contamination: consequences in space-grown wheat. Phytopathology.

[42] Ryba-White M (2001). Growth in microgravity increases susceptibility of soybean to a fungal pathogen. Plant Cell Physiol..

[43] Schuerger AC (2021). Fusarium oxysporum as an Opportunistic Fungal Pathogen on Zinnia hybrida Plants Grown on board the International Space Station. Astrobiology.

[44] Urbaniak C (2018). Draft Genome Sequences of Two Fusarium oxysporum Isolates Cultured from Infected Zinnia hybrida Plants Grown on the International Space Station. Genome Announc..

[45] Zaets I (2011). Bioaugmentation in growing plants for lunar bases. Adv. Space Res..

[46] Wamelink GWW, Frissel JY, Krijnen WHJ, Verwoert MR, Goedhart PW (2014). Can plants grow on Mars and the Moon: a growth experiment on Mars and Moon soil simulants. PLoS ONE.

[47] Paul AL, Elardo SM, Ferl R (2022). Plants grown in Apollo lunar regolith present stress-associated transcriptomes that inform prospects for lunar exploration. Commun. Biol..

[48] Kozyrovska NO (2006). Growing pioneer plants for a lunar base. Adv. Space Res..

[49] Ferl RJ, Paul AL (2010). Lunar plant biology-a review of the Apollo era. Astrobiology.

[50] Brack A, Pillinger CT (1998). Life on Mars: chemical arguments and clues from Martian meteorites. Extremophiles.

[51] Parnell J (2005). Extraction of organic signatures from carbonates and evaporites: from mineral deposits to Mars. Proc. Geologists Assoc..

[52] Müller O (1979). Solar wind nitrogen and indigenous nitrogen in lunar material. Phys. Chem. Earth.

[53] Palomba E (2009). Evidence for Mg-rich carbonates on Mars from a 3.9m absorption feature. Icarus.

[54] Leshin LA (2013). Volatile, isotope, and organic analysis of Martian fines with the Mars curiosity rover. Science.

[55] Mylona P, Pawlowski K, Bisseling T (1995). Symbiotic nitrogen fixation. Plant Cell.

[56] Harris F, Dobbs J, Atkins D, Ippolito JA, Stewart JE (2021). Soil fertility interactions with Sinorhizobium-legume symbiosis in a simulated Martian regolith effects on nitrogen content and plant health. PLoS ONE.

[57] Ralphs M, Franz B, Baker T, Howe S (2015). Water extraction on Mars for an expanding human colony. Life Sci. Space Res..

[58] Maggi F, Pallud C (2010). Martian base agriculture: The effect of low gravity on water flow, nutrient cycles, and microbial biomass dynamics. Adv. Space Res..

[59] Sher Y (2020). Microbial extracellular polysaccharide production and aggregate stability controlled by switchgrass (Panicum virgatum) root biomass and soil water potential. Soil Biol. Biochem..

[60] Nascimento Mdo (2019). Prospects of using biomass of N2-fixing cyanobacteria as an organic fertilizer and soil conditioner. Algal Res..

[61] Abinandan S, Subashchandrabose SR, Venkateswarlu K, Megharaj M (2019). Soil microalgae and cyanobacteria: the biotechnological potential in the maintenance of soil fertility and health. Crit. Rev. Biotechnol..

[62] Li, C. et al. Characteristics of the lunar samples returned by the Chang’E-5 mission. *Natl. Sci. Rev.* **9**, (2022). 10.1093/nsr/nwab188 PMC8974359 35382442

[63] Singh, J. & Kalamdhad, A. Effects of heavy metals on soil, plants, human health and aquatic life. *Int. J. Res. Chem. Environ.* **1**, (2011).

[64] Abbas SZ, Rafatullah M (2021). Recent advances in soil microbial fuel cells for soil contaminants remediation. Chemosphere.

[65] Ayangbenro AS, Babalola OO (2017). A new strategy for heavy metal polluted environments: a review of microbial biosorbents. Int. J. Environ. Res. Public Health.

[66] Wu C (2022). Immobilization of microbes on biochar for water and soil remediation: a review. Environ. Res..

[67] Huang MS, Pan J, Zheng LP (2001). Removal of heavy metals from aqueous solutions using bacteria. J. Shanghai Univ..

[68] González Henao, S. & Ghneim-Herrera, T. Heavy metals in soils and the remediation potential of bacteria associated with the plant microbiome. *Front. Environ. Sci.* **9**, (2021).

[69] Yetunde Mutiat F-B, Gbolahan B, Olu O (2018). A comparative study of the wild and mutatedheavy metal resistant Klebsiella variicola generated for cadmium bioremediation. Bioremediat. J..

[70] Oze C (2021). Perchlorate and agriculture on Mars. Soil Syst..

[71] Eichler A (2021). Challenging the agricultural viability of martian regolith simulants. Icarus.

[72] Wang X (2020). Microbial electrochemistry for bioremediation. Environ. Sci. Ecotechnol..

[73] Sarria M, Gonzales JM, Gerrity D, Batista J (2018). Biological reduction of nitrate and perchlorate in soil microcosms: an electron donor comparison of glycerol, emulsified oil, and mulch extract. Groundw. Monit. Remediation.

[74] Misra G, Smith W, Garner M, Loureiro R (2021). Potential biological remediation strategies for removing perchlorate from Martian regolith. N. Space.

[75] Sunilkumar, U. & LAL, S. Perchlorate reducing bacteria and their insight towards astrobiology. *Int. J. Res. Anal. Rev.* **8**, (2021).

[76] Ewert, M. K. et al. Advanced Life Support Requirements, Assumptions and Reference Missions. in *SAE Technical Paper Series* (SAE International, 2002). 10.4271/2002-01-2480.

[77] Liu H, Yao Z, Fu Y, Feng J (2021). Review of research into bioregenerative life support system(s) which can support humans living in space. Life Sci. Space Res..

[78] Garland, J. Coupling plant growth and waste recycling systems in a controlled life support system (CELSS). *NTRS*, (1992).

[79] Tang Y (2021). Design and establishment of a large-scale controlled ecological life-support system integrated experimental platform. Life Sci. Space Res..

[80] Xie B (2017). The water treatment and recycling in 105-day bioregenerative life support experiment in the Lunar Palace 1. Acta Astronaut..

[81] Tikhomirov AA (2011). Assessment of the possibility of establishing material cycling in an experimental model of the bio-technical life support system with plant and human wastes included in mass exchange. Acta Astronaut..

[82] He W, Liu H, Xing Y, Jones SB (2010). Comparison of three soil-like substrate production techniques for a bioregenerative life support system. Adv. Space Res..

[83] Yu C (2008). Bioconversion of rice straw into a soil-like substrate. Acta Astronaut..

[84] Hendrickx L (2006). Microbial ecology of the closed artificial ecosystem MELiSSA (Micro-Ecological Life Support System Alternative): reinventing and compartmentalizing the Earths food and oxygen regeneration system for long-haul space exploration missions. Res. Microbiol..

[85] Zhu G (2019). Research on the hydrolysis of human urine using biological activated carbon and its application in bioregenerative life support system. Acta Astronaut..

[86] Putnam, D. F. Composition and concentrative properties of human urine. National Aeronautics and Space Administration Contractor Report. Huntington Beach, California: McDonnell Douglas Astronautics Company - Western Division. (1971).

[87] Maggi F, Tang FHM, Pallud C, Gu C (2018). A urine-fuelled soil-based bioregenerative life support system for long-term and long-distance manned space missions. Life Sci. Space Res..

[88] Subbarao, G., Yorio, N., Wheeler, R. & Stutte, G. Plant Growth and Human Life Support for Space Travel. In *Handbook of Plant and Crop Physiology* (CRC Press, 2001). 10.1201/9780203908426.ch48.

[89] Garland JL, Levine LH, Yorio NC, Hummerick ME (2004). Response of graywater recycling systems based on hydroponic plant growth to three classes of surfactants. Water Res..

[90] Garland J (2000). Graywater processing in recirculating hydroponic systems: phytotoxicity, surfactant degradation, and bacterial dynamics. Water Res..

[91] Horneck G (2003). Humex, a study on the survivability and adaptation of humans to long-duration exploratory missions, part I: Lunar missions. Adv. Space Res..

[92] Rosgaard L, de Porcellinis AJ, Jacobsen JH, Frigaard N-U, Sakuragi Y (2012). Bioengineering of carbon fixation, biofuels, and biochemicals in cyanobacteria and plants. J. Biotechnol..

[93] Zahra Z, Choo DH, Lee H, Parveen A (2020). Cyanobacteria: review of current potentials and applications. Environments.

[94] Rasmussen B, Fletcher IR, Brocks JJ, Kilburn MR (2008). Reassessing the first appearance of eukaryotes and cyanobacteria. Nature.

[95] Gan F, Bryant DA (2015). Adaptive and acclimative responses of cyanobacteria to far-red light. Environ. Microbiol..

[96] Gan F (2014). Extensive remodeling of a cyanobacterial photosynthetic apparatus in far-red light. Science.

[97] Gisriel CJ (2022). Structure of a photosystem I-ferredoxin complex from a marine cyanobacterium provides insights into far-red light photoacclimation. J. Biol. Chem..

[98] Rabbow E (2017). EXPOSE-R2: The Astrobiological ESA Mission on Board of the International Space Station. Front. Microbiol..

[99] Rabbow E (2009). EXPOSE, an astrobiological exposure facility on the international space station - from proposal to flight. Orig. Life Evol. Biospheres.

[100] Billi D (2019). A desert cyanobacterium under simulated Mars-like conditions in low earth orbit: implications for the habitability of Mars. Astrobiology.

[101] Fleming ED, Bebout BM, Tan MX, Selch F, Ricco AJ (2014). Biological system development for GraviSat: A new platform for studying photosynthesis and microalgae in space. Life Sci. Space Res..

[102] Gòdia F (2002). MELISSA: a loop of interconnected bioreactors to develop life support in Space. J. Biotechnol..

[103] Poughon, L., Creuly, C., Godia, F., Leys, N. & Dussap, C.-G. Photobioreactor Limnospira indica growth model: application from the MELiSSA plant pilot scale to ISS flight experiment. *Front. Astron. Space Sci.* **8**, (2021).

[104] Detrell, G. Chlorella vulgaris photobioreactor for oxygen and food production on a Moon basepotential and challenges. *Front. Astron. Space Sci.* **8**, (2021).

[105] Häder D (2020). On the way to Mars flagellated algae in bioregenerative life support systems under microgravity conditions. Front. Plant Sci..

[106] Sachdeva, N. et al. Ground demonstration of the use of limnospira indica for air revitalization in a bioregenerative life-support system setup: effect of non-nitrified urinederived nitrogen sources. *Front. Astron. Space Sci.* **8**, (2021).

[107] Poughon L (2020). Limnospira indica PCC8005 growth in photobioreactor: model and simulation of the ISS and ground experiments. Life Sci. Space Res..

[108] Kyazze, G. Four ways microbial fuel cells might revolutionise electricity production in the future. *The Conversation* https://theconversation.com/four-ways-microbial-fuel-cells-might-revolutionise-electricity-production-in-the-future-152184 (2020).

[109] Logan, B. E. *Microbial Fuel Cells*. (John Wiley & Sons, Inc., 2007).

[110] Arkatkar A, Mungray AK, Sharma P (2021). Study of electrochemical activity zone of *Pseudomonas aeruginosa* in microbial fuel cell. Process Biochem..

[111] Lin T (2016). Synthetic Saccharomyces cerevisiae - Shewanella oneidensis consortium enables glucose-fed high-performance microbial fuel cell. AIChE J..

[112] Kondaveeti S, Lee S-H, Park H-D, Min B (2020). Specific enrichment of different Geobacter sp. in anode biofilm by varying interspatial distance of electrodes in air-cathode microbial fuel cell (MFC). Electrochim. Acta.

[113] Vasyliv, O. M., Maslovska, O. D., Ferensovych, Y. P., Bilyy, O. I. & Hnatush, S. O. Interconnection between tricarboxylic acid cycle and energy generation in microbial fuel cell performed by desulfuromonas acetoxidans IMV B-7384. in *Energy Harvesting and Storage: Materials, Devices, and Applications VI* (eds. Dhar, N. K. & Dutta, A. K.) (SPIE, 2015).

[114] Ren H, Tian H, Gardner CL, Ren T-L, Chae J (2016). A miniaturized microbial fuel cell with three-dimensional graphene macroporous scaffold anode demonstrating a record power density of over 100.167em000 W msup-3/sup. Nanoscale.

[115] Trapero JR, Horcajada L, Linares JJ, Lobato J (2017). Is microbial fuel cell technology ready? An economic answer towards industrial commercialization. Appl. Energy.

[116] Potter, M. C. Electrical effects accompanying the decomposition of organic compounds. *Proc. R. Soc. Lond. Series B, Containing Papers of a Biological Character* **84**, 260–276 (1911).

[117] Zhang J (2019). Life cycle assessment of osmotic microbial fuel cells for simultaneous wastewater treatment and resource recovery. Int J. Life Cycle Assess..

[118] Cao X, Song H, Yu C, Li X (2015). Simultaneous degradation of toxic refractory organic pesticide and bioelectricity generation using a soil microbial fuel cell. Bioresour. Technol..

[119] Bose D, Santra M, Sanka RVSP, Krishnakumar B (2020). Bioremediation analysis of sediment microbial fuel cells for energy recovery from microbial activity in soil. Int. J. Energy Res..

[120] Ieropoulos I, Greenman J, Melhuish C (2010). Improved energy output levels from small-scale microbial fuel cells. Bioelectrochemistry.

[121] Linares RV (2019). Scale up of microbial fuel cell stack system for residential wastewater treatment in continuous mode operation. Water.

[122] de Vet SJ, Rutgers R (2007). From waste to energy: First experimental bacterial fuel cells onboard the international space station. Microgravity Sci. Technol..

[123] Electrified Bacteria Clean Wastewater, Generate Power | NASA Spinoff. https://spinoff.nasa.gov/Spinoff2019/ee_1.html.

[124] Cid CA, Stinchcombe A, Ieropoulos I, Hoffmann MR (2018). Urine microbial fuel cells in a semi-controlled environment for onsite urine pre-treatment and electricity production. J. Power Sources.

[125] Lu S (2019). Resource recovery microbial fuel cells for urine-containing wastewater treatment without external energy consumption. Chem. Eng. J..

[126] Yang N, Liu H, Jin X, Li D, Zhan G (2020). One-pot degradation of urine wastewater by combining simultaneous halophilic nitrification and aerobic denitrification in air-exposed biocathode microbial fuel cells (AEB-MFCs). Sci. Total Environ..

[127] Ieropoulos I, Greenman J, Melhuish C (2012). Urine utilisation by microbial fuel cells energy fuel for the future. Phys. Chem. Chem. Phys..

[128] Cao X (2009). A new method for water desalination using microbial desalination. Cells Environ. Sci. Amp Technol..

[129] Fangzhou D, Zhenglong L, Shaoqiang Y, Beizhen X, Hong L (2011). Electricity generation directly using human feces wastewater for life support system. Acta Astronaut..

[130] Colombo A (2017). Signal trends of microbial fuel cells fed with different food-industry residues. Int. J. Hydrog. Energy.

[131] Gajda I (2018). Miniaturized ceramic-based microbial fuel cell for efficient power generation from urine and stack development. Front. Energy Res..

[132] Gajda I, Obata O, Salar-Garcia MJ, Greenman J, Ieropoulos IA (2020). Long-term bio-power of ceramic microbial fuel cells in individual and stacked configurations. Bioelectrochemistry.

[133] Ieropoulos IA (2016). Pee power urinal microbial fuel cell technology field trials in the context of sanitation. Environ. Sci.: Water Res. Amp. Technol..

[134] Jones DL, Nguyen C, Finlay RD (2009). Carbon flow in the rhizosphere: carbon trading at the soilroot interface. Plant Soil.

[135] Schamphelaire Lde (2008). Microbial fuel cells generating electricity from rhizodeposits of rice. Plants Environ. Sci. Amp. Technol..

[136] Lee, R. & Miller, A. A novel approach to harvesting energy from agriculture in microbe-polluted water: the implementation of plant microbial fuel cells in hydroponic chambers. *Columbia Junior Sci. J.* (2018).

[137] Habibul N, Hu Y, Sheng GP (2016). Microbial fuel cell driving electrokinetic remediation of toxic metal contaminated soils. J. Hazard Mater..

[138] Bradley RW, Bombelli P, Lea-Smith DJ, Howe CJ (2013). Terminal oxidase mutants of the cyanobacterium Synechocystis sp. PCC 6803 show increased electrogenic activity in biological photo-voltaic systems. Phys. Chem. Chem. Phys..

[139] MeiRong M, LiMin C, XiaoFang Y, ZongWu D (2012). Study on the performance of photosynthetic microbial fuel cells powered by synechocystis PCC-6803. Kezaisheng Nengyuan / Renew. Energy Resour..

[140] Wenzel T, Härtter D, Bombelli P, Howe CJ, Steiner U (2018). Porous translucent electrodes enhance current generation from photosynthetic biofilms. Nat. Commun..

[141] Dawar S, Behera BK, Mohanty P (1998). Development of a low-cost oxy-hydrogen bio-fuel cell for generation of electricity using Nostoc as a source of hydrogen. Int. J. Energy Res..

[142] Güttler J (2020). Direct electron transport as a possible mechanism of electrogenic activity across a range of benthic cyanobacteria in a photosynthetic microbial fuel cell. N. Z. J. Bot..

[143] Pisciotta JM, Zou Y, Baskakov IV (2011). Role of the photosynthetic electron transfer chain in electrogenic activity of cyanobacteria. Appl. Microbiol. Biotechnol..

[144] Oluyide OO (2020). Effect of some operational conditions on bioelectricity production in algal fuel cell. Int. J. Renew. Energy Technol..

[145] Maity JP (2014). The production of biofuel and bioelectricity associated with wastewater treatment by green algae. Energy.

[146] Kaushik S, Sarma MK, Goswami P (2017). FRET-guided surging of cyanobacterial photosystems improves and stabilizes current in photosynthetic microbial fuel cell. J. Mater. Chem. A Mater..

[147] Wall, M. Nuclear Reactor for Mars Outpost Could Be Ready to Fly by 2022 | Space. *Space.com* https://www.space.com/nuclear-reactor-for-mars-outpost-2022.html (2019).

[148] Voutsinos, M. Biomining the elements of the future. *The Conversation* https://theconversation.com/biomining-the-elements-of-the-future-87621 (2018).

[149] Olson GJ, Sakai CK, Parks EJ, Brinckman FE (1990). Bioleaching of cobalt from smelter wastes by Thiobacillus ferrooxidans. J. Ind. Microbiol..

[150] Giaveno A, Lavalle L, Chiacchiarini P, Donati E (2007). Bioleaching of zinc from low-grade complex sulfide ores in an airlift by isolated Leptospirillum ferrooxidans. Hydrometallurgy.

[151] Clark DA, Norris PR (1996). Acidimicrobium ferrooxidans gen. nov., sp. nov.: mixed-culture ferrous iron oxidation with Sulfobacillus species. Microbiol. (N. Y).

[152] Rawlings DE (2005). Characteristics and adaptability of iron- and sulfur-oxidizing microorganisms used for the recovery of metals from minerals and their concentrates. Micro. Cell Fact..

[153] Solisio C, Lodi A, Veglio F (2002). Bioleaching of zinc and aluminium from industrial waste sludges by means of Thiobacillus ferrooxidans. Waste Manag..

[154] Pronk JT, Johnson DB (1992). Oxidation and reduction of iron by acidophilic bacteria. Geomicrobiol. J..

[155] Deveci H, Akcil A, Alp I (2004). Bioleaching of complex zinc sulphides using mesophilic and thermophilic bacteria: comparative importance of pH and iron. Hydrometallurgy.

[156] McSween HY, Taylor GJ, Wyatt MB (2009). Elemental composition of the martian crust. Science.

[157] Ruzicka A, Snyder GA, Taylor LA (2001). Comparative geochemistry of basalts from the moon, earth, HED asteroid, and Mars: implications for the origin of the moon. Geochim Cosmochim. Acta.

[158] Doody, D. *Deep Space Craft* (Springer Berlin Heidelberg, 2009).

[159] Loudon C-M (2017). BioRock: new experiments and hardware to investigate microbemineral interactions in space. Int. J. Astrobiol..

[160] Cockell CS (2020). Space station biomining experiment demonstrates rare earth element extraction in microgravity and Mars gravity. Nat. Commun..

[161] Cockell, C. S. et al. Microbially-enhanced vanadium mining and bioremediation under micro- and Mars gravity on the international space station. *Front. Microbiol.* **12** (2021). 10.3389/fmicb.2021.641387 PMC8047202 33868198

[162] Yin S, Wang L, Wu A, Free ML, Kabwe E (2018). Enhancement of copper recovery by acid leaching of high-mud copper oxides: a case study at Yangla Copper Mine, China. J. Clean. Prod..

[163] Cook, R. T. Methane heat transfer investigation. Contractor Report. https://ntrs.nasa.gov/citations/19850004010 (1984).

[164] Harbaugh, J. NASA 3-D Prints First Full-Scale Copper Rocket Engine Part. (2015).

[165] Sonter MJ (1997). The technical and economic feasibility of mining the near-earth asteroids. Acta Astronaut..

[166] Busch M (2004). Profitable asteroid mining. JBIS.

[167] Kryzanowski, T. & Mardon, A. Mining potential of asteriod belt. *Can. Min. J.* **111**, (1990).

[168] Levin, G. v, Kuznetz, L. & Lafleur, A. L. Approaches to resolving the question of life on Mars. in *SPIE Proceedings* (ed. Hoover, R. B.) (SPIE, 2000). 10.1117/12.411620.

[169] Chakarvarty, U. Renewable Energy Materials Supply Implications. *Association for Energy Economics, Energy Forum* **37** (2018).

[170] Goonan, T. G. Rare earth elements: end use and recyclability. 10.3133/sir20115094 (2011).

[171] Rawlings, D. E. & Johnson, D. B. *Biomining*. *Biomining* (Springer-Verlag Berlin Heidelberg, 2007).

[172] Volger R (2020). Theoretical bioreactor design to perform microbial mining activities on mars. Acta Astronaut..

[173] Khalil AS, Collins JJ (2010). Synthetic biology: applications come of age. Nat. Rev. Genet..

[174] Tesei D, Jewczynko A, Lynch A, Urbaniak C (2022). Understanding the complexities and changes of the astronaut microbiome for successful long-duration space missions. Life.

[175] Voorhies AA (2019). Study of the impact of long-duration space missions at the International Space Station on the astronaut microbiome. Sci. Rep..

[176] Baevsky RM (2007). Autonomic cardiovascular and respiratory control during prolonged spaceflights aboard the International Space Station. J. Appl Physiol..

[177] Parsons-Wingerter P, Hosamani R, Vickerman MB, Bhattacharya S (2015). Mapping by VESGEN of wing vein phenotype in Drosophila for quantifying adaptations to space environments. Gravit. Space Res..

[178] Voorhies, A. A. & Lorenzi, H. A. The challenge of maintaining a healthy microbiome during long-duration space missions. *Front. Astron. Space Sci.* **3**, (2016).

[179] Crucian B, Sams C (2009). Immune system dysregulation during spaceflight: clinical risk for exploration-class missions. J. Leukoc. Biol..

[180] Turroni S (2020). Gut microbiome and space travelers’ health: state of the art and possible pro/prebiotic strategies for long-term space missions. Front. Physiol..

[181] Douglas GL, Voorhies AA (2017). Evidence based selection of probiotic strains to promote astronaut health or alleviate symptoms of illness on long duration spaceflight missions. Benef. Microbes.

[182] Islam SU (2016). Clinical uses of probiotics. Medicine.

[183] Ritchie LE (2015). Space environmental factor impacts upon murine colon microbiota and mucosal homeostasis. PLoS ONE.

[184] Smirnov KV, Lizko NN (1987). Problems of space gastroenterology and microenvironment. Food / Nahr..

[185] Shao D (2016). Simulated microgravity affects some biological characteristics of Lactobacillus acidophilus. Appl. Microbiol. Biotechnol..

[186] Sakai T (2018). Probiotics into outer space: feasibility assessments of encapsulated freeze-dried probiotics during 1 month’s storage on the International Space Station. Sci. Rep..

[187] Fajardo-Cavazos P, Nicholson WL (2021). Shelf life and simulated gastrointestinal tract survival of selected commercial probiotics during a simulated round-trip journey to Mars. Front. Microbiol..

[188] Simon Á, Smarandache A, Iancu V, Pascu ML (2021). Stability of antimicrobial drug molecules in different gravitational and radiation conditions in view of applications during outer space missions. Molecules.

[189] Soga S (2001). Stereospecific antitumor activity of radicicol oxime derivatives. Cancer Chemother. Pharm..

[190] Lam KS (1998). The effects of space flight on the production of monorden by Humicola fuscoatra WC5157 in solid-state fermentation. Appl. Microbiol. Biotechnol..

[191] Lam KS (2002). The effect of space flight on the production of actinomycin D by Streptomyces plicatus. J. Ind. Microbiol. Biotechnol..

[192] Benoit MR (2006). Microbial antibiotic production aboard the International Space Station. Appl. Microbiol. Biotechnol..

[193] Schei K (2017). Early gut mycobiota and mother-offspring transfer. Microbiome.

[194] Angulo M, Reyes-Becerril M, Medina-Córdova N, Tovar-Ramirez D, Angulo C (2020). Probiotic and nutritional effects of Debaryomyces hansenii on animals. Appl. Microbiol. Biotechnol..

[195] Zeng A (2019). Effects of Debaryomyces hansenii treatment on intestinal mucosa microecology in mice with antibiotic-associated diarrhea. PLoS ONE.

[196] Wu Y, Tang Y, Xiao N-Q, Wang C-H, Tan Z-J (2020). Bacterial lactase gene characteristics in intestinal contents of antibiotic-associated diarrhea mice treated with Debaryomyces hansenii. Med. Sci. Monit..

[197] He Y (2019). Influence of Debaryomyces hansenii on bacterial lactase gene diversity in intestinal mucosa of mice with antibiotic-associated diarrhea. PLoS ONE.

[198] Rizzatti G, Lopetuso LR, Gibiino G, Binda C, Gasbarrini A (2017). Proteobacteria: a common factor in human diseases. Biomed. Res. Int..

[199] Urbaniak C (2020). The influence of spaceflight on the astronaut salivary microbiome and the search for a microbiome biomarker for viral reactivation. Microbiome.

[200] Claesen J, Fischbach MA (2014). Synthetic microbes as drug delivery systems. ACS Synth. Biol..

[201] Aggarwal N, Breedon AME, Davis CM, Hwang IY, Chang MW (2020). Engineering probiotics for therapeutic applications: recent examples and translational outlook. Curr. Opin. Biotechnol..

[202] Zhou Z (2020). Engineering probiotics as living diagnostics and therapeutics for improving human health. Micro. Cell Fact..

[203] Piñero-Lambea C, Ruano-Gallego D, Fernández LÁ (2015). Engineered bacteria as therapeutic agents. Curr. Opin. Biotechnol..

[204] O’Toole PW, Marchesi JR, Hill C (2017). Next-generation probiotics: the spectrum from probiotics to live biotherapeutics. Nat. Microbiol..

[205] Garrido V (2021). Engineering a genome-reduced bacterium to eliminate *Staphylococcus aureus* biofilms in vivo. Mol. Syst. Biol..

[206] Neil K (2021). High-efficiency delivery of CRISPR-Cas9 by engineered probiotics enables precise microbiome editing. Mol. Syst. Biol..

[207] Strucko T, Andersen NL, Mahler MR, Martinez JL, Mortensen UH (2021). A CRISPR/Cas9 method facilitates efficient oligo-mediated gene editing in Debaryomyces hansenii. Synth. Biol..

[208] Banjara N, Nickerson KW, Suhr MJ, Hallen-Adams HE (2016). Killer toxin from several food-derived Debaryomyces hansenii strains effective against pathogenic Candida yeasts. Int. J. Food Microbiol..

[209] Al-Qaysi SAS, Al-Haideri H, Thabit ZA, Al-Kubaisy WHAA-R, Ibrahim JAA-R (2017). Production, characterization, and antimicrobial activity of mycocin produced by Debaryomyces hanseni DSMZ70238. Int. J. Microbiol..

[210] Hwang IY (2013). Reprogramming microbes to be pathogen-seeking killers. ACS Synth. Biol..

[211] Saeidi N (2011). Engineering microbes to sense and eradicate Pseudomonas aeruginosa a human pathogen. Mol. Syst. Biol..

[212] Hwang IY (2017). Engineered probiotic Escherichia coli can eliminate and prevent Pseudomonas aeruginosa gut infection in animal models. Nat. Commun..

[213] Geldart KG (2018). Engineered scp E. coli scp Nissle 1917 for the reduction of vancomycin-resistant Enterococcus in the intestinal tract. Bioeng. Amp Transl. Med..

[214] Tscherner, M., Giessen, T. W., Markey, L., Kumamoto, C. A. & Silver, P. A. A synthetic system that senses *Candida albicans* and inhibits virulence factors 10.1101/342287 (2018). 10.1021/acssynbio.8b00457 30608638

[215] Plavec TV (2019). Engineered Lactococcus lactis secreting IL-23 receptor-targeted REX protein blockers for modulation of IL-23/Th17-mediated inflammation. Microorganisms.

[216] Lubkowicz D (2018). Reprogramming probiotic *Lactobacillus reuteri* as a biosensor for *Staphylococcus aureus* derived AIP-I detection. ACS Synth. Biol..

[217] McFarland LV, Evans CT, Goldstein EJC (2018). Strain-specificity and disease-specificity of probiotic efficacy: a systematic review and meta-analysis. Front Med (Lausanne).

[218] Scott BM (2021). Self-tunable engineered yeast probiotics for the treatment of inflammatory bowel disease. Nat. Med..

[219] Archer EJ, Robinson AB, Süel GM (2012). Engineered *E. coli* that detect and respond to gut inflammation through nitric oxide sensing. ACS Synth. Biol..

[220] Daeffler KN-M (2017). Engineering bacterial thiosulfate and tetrathionate sensors for detecting gut inflammation. Mol. Syst. Biol..

[221] Kotula JW (2014). Programmable bacteria detect and record an environmental signal in the mammalian gut. Proc. Natl Acad. Sci. USA.

[222] Çiftçioǧlu N, Haddad RS, Golden DC, Morrison DR, McKay DS (2005). A potential cause for kidney stone formation during space flights: enhanced growth of nanobacteria in microgravity. Kidney Int..

[223] Whitson, P., Pietrzyk, R., Jones, J. & Sams, C. Renal stone assessment during spaceflight - Assessment and countermeausure validation. in *2001 Conference and Exhibit on International Space Station Utilization* (American Institute of Aeronautics and Astronautics, 2001).

[224] Pietrzyk, R., Jones, J., Sams, C. & Whitson, P. Renal stone formation among astronauts - PubMed. *Aviat. Space Environ. Med.* **78**, (2007). 17511294

[225] Siener R (2013). The role of Oxalobacter formigenes colonization in calcium oxalate stone disease. Kidney Int..

[226] Al KF (2020). Oxalate-degrading Bacillus subtilis mitigates urolithiasis in a Drosophila melanogaster model. mSphere.

[227] Grujic D (2008). Hyperoxaluria is reduced and nephrocalcinosis prevented with an oxalate-degrading enzyme in mice with hyperoxaluria. Am. J. Nephrol..

[228] Sasikumar P (2014). Recombinant Lactobacillus plantarum expressing and secreting heterologous oxalate decarboxylase prevents renal calcium oxalate stone deposition in experimental rats. J. Biomed. Sci..

[229] Ettinger G, MacDonald K, Reid G, Burton JP (2014). The influence of the human microbiome and probiotics on cardiovascular health. Gut Microbes.

[230] Gan XT (2014). Probiotic administration attenuates myocardial hypertrophy and heart failure after myocardial infarction in the rat. Circ. Heart Fail.

[231] Naruszewicz M, Johansson M-L, Zapolska-Downar D, Bukowska H (2002). Effect of Lactobacillus plantarum 299v on cardiovascular disease risk factors in smokers. Am. J. Clin. Nutr..

[232] Chen Z (2014). Incorporation of therapeutically modified bacteria into gut microbiota inhibits obesity. J. Clin. Invest..

[233] Hughson RL, Helm A, Durante M (2017). Heart in space: effect of the extraterrestrial environment on the cardiovascular system. Nat. Rev. Cardiol..

[234] Meerman M (2021). Myocardial disease and long-distance space travel: solving the radiation problem. Front. Cardiovasc. Med..

[235] Trolio R, di, Lorenzo G, di, Fumo B, Ascierto PA (2015). Cosmic radiation and cancer: is there a link?. Future Oncol..

[236] Durante M, Cucinotta FA (2008). Heavy ion carcinogenesis and human space exploration. Nat. Rev. Cancer.

[237] Edmondson EF (2020). Genomic mapping in outbred mice reveals overlap in genetic susceptibility for HZE ion and -rayinduced tumors. Sci. Adv..

[238] Zhou S, Gravekamp C, Bermudes D, Liu K (2018). Tumour-targeting bacteria engineered to fight cancer. Nat. Rev. Cancer.

[239] Zhu H (2011). Antitumor effect of sFlt-1 gene therapy system mediated by Bifidobacterium Infantis on Lewis lung cancer in mice. Cancer Gene Ther..

[240] Piñero-Lambea C (2014). Programming controlled adhesion of *E. coli* to target surfaces, cells, and tumors with synthetic adhesins. ACS Synth. Biol..

[241] Chowdhury S (2019). Programmable bacteria induce durable tumor regression and systemic antitumor immunity. Nat. Med..

[242] Yang H (2021). Genetically engineered bacterial protein nanoparticles for targeted cancer therapy. Int. J. Nanomed..

[243] Agrawal N (2004). Bacteriolytic therapy can generate a potent immune response against experimental tumors. Proc. Natl Acad. Sci. USA.

[244] Feng X (2020). Novel insights into the role of Clostridium novyi-NT related combination bacteriolytic therapy in solid tumors (Review). Oncol. Lett..

[245] Badie F (2021). Use of Salmonella bacteria in cancer therapy: direct, drug delivery and combination approaches. Front. Oncol..

[246] Wang Y, Chen J, Tang BO, Zhang X, Hua Z-C (2013). Systemic administration of attenuated Salmonella typhimurium in combination with interleukin-21 for cancer therapy. Mol. Clin. Oncol..

[247] Yoon W, Yoo Y, Chae YS, Kee S-H, Kim BM (2018). Therapeutic advantage of genetically engineered Salmonella typhimurium carrying short hairpin RNA against inhibin alpha subunit in cancer treatment. Ann. Oncol..

[248] Toso JF (2002). Phase I study of the intravenous administration of attenuated Salmonella typhimurium to patients with metastatic melanoma. J. Clin. Oncol..

[249] Nemunaitis J (2003). Pilot trial of genetically modified, attenuated Salmonella expressing the *E. coli* cytosine deaminase gene in refractory cancer patients. Cancer Gene Ther..

[250] Maletzki C, Linnebacher M, Kreikemeyer B, Emmrich J (2007). Pancreatic cancer regression by intratumoural injection of live Streptococcus pyogenes in a syngeneic mouse model. Gut.

[251] Danino T (2015). Programmable probiotics for detection of cancer in urine. Sci. Transl. Med..

[252] Alvarez AL, Weyers SL, Goemann HM, Peyton BM, Gardner RD (2021). Microalgae, soil and plants: a critical review of microalgae as renewable resources for agriculture. Algal Res..

[253] Torres-Tiji Y, Fields FJ, Mayfield SP (2020). Microalgae as a future food source. Biotechnol. Adv..

[254] Olsson-Francis K, Cockell CS (2010). Use of cyanobacteria for in-situ resource use in space applications. Planet Space Sci..

[255] Brown, M. Curious Kids: Where does the oxygen come from in the International Space Station, and why don’t they run out of air? *The Conversation* https://theconversation.com/curious-kids-where-does-the-oxygen-come-from-in-the-international-space-station-and-why-dont-they-run-out-of-air-82910 (2017).

[256] Berepiki A, Hitchcock A, Moore CM, Bibby TS (2016). Tapping the unused potential of photosynthesis with a heterologous electron sink. ACS Synth. Biol..

[257] Santos-Merino M (2021). Improved photosynthetic capacity and photosystem I oxidation via heterologous metabolism engineering in cyanobacteria. Proc. Natl Acad. Sci. USA.

[258] Kamennaya NA (2015). Installing extra bicarbonate transporters in the cyanobacterium Synechocystis sp. PCC6803 enhances biomass production. Metab. Eng..

[259] Sakai M, Ogawa T, Matsuoka M, Fukuda H (1997). Photosynthetic conversion of carbon dioxide to ethylene by the recombinant cyanobacterium, Synechococcus sp. PCC 7942, which harbors a gene for the ethylene-forming enzyme of Pseudomonas syringae. J. Ferment. Bioeng..

[260] Lindberg P, Park S, Melis A (2010). Engineering a platform for photosynthetic isoprene production in cyanobacteria, using Synechocystis as the model organism. Metab. Eng..

[261] Atsumi S, Higashide W, Liao JC (2009). Direct photosynthetic recycling of carbon dioxide to isobutyraldehyde. Nat. Biotechnol..

[262] Deng M-D, Coleman JR (1999). Ethanol synthesis by genetic engineering in cyanobacteria. Appl. Environ. Microbiol..

[263] Dogutan DK, Nocera DG (2019). Artificial photosynthesis at efficiencies greatly exceeding that of natural photosynthesis. Acc. Chem. Res..

[264] Wells ML (2016). Algae as nutritional and functional food sources: revisiting our understanding. J. Appl. Phycol..

[265] Becker EW (2007). Micro-algae as a source of protein. Biotechnol. Adv..

[266] Kroth PG (2018). Genome editing in diatoms: achievements and goals. Plant Cell Rep..

[267] Slattery SS (2020). Plasmid-based complementation of large deletions in Phaeodactylum tricornutum biosynthetic genes generated by Cas9 editing. Sci. Rep..

[268] Karas BJ (2015). Designer diatom episomes delivered by bacterial conjugation. Nat. Commun..

[269] Gale GAR (2019). Emerging species and genome editing tools: future prospects in Cyanobacterial Synthetic Biology. Microorganisms.

[270] Serif M (2018). One-step generation of multiple gene knock-outs in the diatom Phaeodactylum tricornutum by DNA-free genome editing. Nat. Commun..

[271] Behler J, Vijay D, Hess WR, Akhtar MK (2018). CRISPR-based technologies for metabolic engineering in cyanobacteria. Trends Biotechnol..

[272] Cochrane RR (2020). Rapid method for generating designer algal mitochondrial genomes. Algal Res..

[273] Slattery SS (2018). An expanded plasmid-based genetic toolbox enables Cas9 genome editing and stable maintenance of synthetic pathways in Phaeodactylum tricornutum. ACS Synth. Biol..

[274] Koo KM (2017). The mechanism of starch over-accumulation in Chlamydomonas reinhardtii high-starch mutants identified by comparative transcriptome analysis. Front. Microbiol..

[275] Baek K (2017). Photoautotrophic production of macular pigment in a Chlamydomonas reinhardtii strain generated by using DNA-free CRISPR-Cas9 RNP-mediated mutagenesis. Biotechnol. Bioeng..

[276] Lee AG (2020). Spaceflight associated neuro-ocular syndrome (SANS) and the neuro-ophthalmologic effects of microgravity: a review and an update. npj Microgravity.

[277] Hindupur, A. et al. BioNutrients-1: On-Demand Production of Nutrients in Space. NASA government report. https://ntrs.nasa.gov/citations/20190033398 (2019).

[278] Durante M, Cucinotta FA (2011). Physical basis of radiation protection in space travel. Rev. Mod. Phys..

[279] Ball, N. et al. BioNutrients-2: Improvements to the BioNutrients-1 Nutrient Production System. *50th International Conference on Environmental Systems*, (2021).

[280] Snyder JE, Walsh D, Carr PA, Rothschild LJ (2019). A makerspace for life support systems in space. Trends Biotechnol..

[281] KISTLER SS (1931). Coherent expanded aerogels and jellies. Nature.

[282] Paulauskiene T, Uebe J, Ziogas M (2021). Cellulose aerogel composites as oil sorbents and their regeneration. PeerJ.

[283] Soleimani Dorcheh A, Abbasi MH (2008). Silica aerogel; synthesis, properties and characterization. J. Mater. Process. Tech..

[284] Wordsworth R, Kerber L, Cockell C (2019). Enabling Martian habitability with silica aerogel via the solid-state greenhouse effect. Nat. Astron.

[285] Zhao S (2020). Additive manufacturing of silica aerogels. Nature.

[286] Jones, S. M. & Sakamoto, J. Applications of Aerogels in Space Exploration. in *Aerogels Handbook* 721–746 (Springer New York, 2011). 10.1007/978-1-4419-7589-8_32.

[287] Yang Y (2005). Toughness of spider silk at high and low temperatures. Adv. Mater..

[288] Bowen CH (2019). Seeded chain-growth polymerization of proteins in living bacterial cells. ACS Synth. Biol..

[289] Voigt CA (2020). Synthetic biology 20202030: six commercially-available products that are changing our world. Nat. Commun..

[290] Roberts AD (2019). Synthetic biology for fibers, adhesives, and active camouflage materials in protection and aerospace. MRS Commun..

[291] Kim E (2021). A biosynthetic hybrid spidroin-amyloid-mussel foot protein for underwater adhesion on diverse surfaces. ACS Appl Mater. Interfaces.

[292] Dubbin K (2021). Projection microstereolithographic microbial bioprinting for engineered biofilms. Nano Lett..

[293] Di Martino P (2018). Extracellular polymeric substances, a key element in understanding biofilm phenotype. AIMS Microbiol..

[294] Akiyama H (2011). Antibiotics-free stable polyhydroxyalkanoate (PHA) production from carbon dioxide by recombinant cyanobacteria. Bioresour. Technol..

[295] Osanai T (2013). Increased bioplastic production with an RNA polymerase sigma factor SigE during nitrogen starvation in Synechocystis sp. PCC 6803. DNA Res..

[296] Sudesh K, Taguchi K, Doi Y (2002). Effect of increased PHA synthase activity on polyhydroxyalkanoates biosynthesis in Synechocystis sp. PCC6803. Int J. Biol. Macromol..

[297] Averesch NJH, Rothschild LJ (2019). Metabolic engineering of Bacillus subtilis for production of para-aminobenzoic acid unexpected importance of carbon source is an advantage for space application. Micro. Biotechnol..

[298] Averesch NJH, Winter G, Krömer JO (2016). Production of para-aminobenzoic acid from different carbon-sources in engineered Saccharomyces cerevisiae. Micro. Cell Fact..

[299] Prater T (2018). 3D Printing in Zero G Technology Demonstration Mission: complete experimental results and summary of related material modeling efforts. Int. J. Adv. Manuf. Technol..

[300] Gu J-D (2003). Microbiological deterioration and degradation of synthetic polymeric materials: recent research advances. Int. Biodeterior. Amp. Biodegrad..

[301] Tesei D (2022). Black fungi research: out-of-this-world implications. Encyclopedia.

[302] Webb JS (2000). Fungal colonization and biodeterioration of plasticized polyvinyl chloride. Appl. Environ. Microbiol..

[303] Tesei D (2020). Shotgun proteomics reveals putative polyesterases in the secretome of the rock-inhabiting fungus Knufia chersonesos. Sci. Rep..

[304] Tesei D (2021). Effects of simulated microgravity on the proteome and secretome of the polyextremotolerant black fungus Knufia chersonesos. Front. Genet..

[305] Dadachova E, Casadevall A (2008). Ionizing radiation: how fungi cope, adapt, and exploit with the help of melanin. Curr. Opin. Microbiol..

[306] Blachowicz A (2019). Proteomic and metabolomic characteristics of extremophilic fungi under simulated mars conditions. Front. Microbiol..

[307] Zakharova K, Marzban G, de Vera J-P, Lorek A, Sterflinger K (2014). Protein patterns of black fungi under simulated Mars-like conditions. Sci. Rep..

[308] Atanasova N, Stoitsova S, Paunova-Krasteva T, Kambourova M (2021). Plastic degradation by extremophilic bacteria. Int. J. Mol. Sci..

[309] Reisz JA, Bansal N, Qian J, Zhao W, Furdui CM (2014). Effects of ionizing radiation on biological molecules mechanisms of damage and emerging methods of detection. Antioxid. Amp. Redox Signal..

[310] Azzam EI, Jay-Gerin J-P, Pain D (2012). Ionizing radiation-induced metabolic oxidative stress and prolonged cell injury. Cancer Lett..

[311] Cucinotta FA, Kim M-HY, Willingham V, George KA (2008). Physical and biological organ dosimetry analysis for international space station astronauts. Radiat. Res..

[312] American Nuclear Society -- ANS. https://www.ans.org/.

[313] Naito M (2020). Radiation dose and its protection in the Moon from galactic cosmic rays and solar energetic particles: at the lunar surface and in a lava tube. J. Radiol. Prot..

[314] Mendell, W. *Lunar Bases and Space Activities of the 21st Century*. (1985).

[315] Aziz, Md AB, Rahman MdF, Prodhan MdMH (2018). Comparison of lead, copper and aluminium as gamma radiation shielding material through experimental measurements and simulation using MCNP version 4c. Int. J. Contemp. Res. Rev..

[316] Zhdanova NN, Tugay T, Dighton J, Zheltonozhsky V, Mcdermott P (2004). Ionizing radiation attracts soil fungi. Mycol. Res..

[317] Vember VV, Zhdanova NN (2001). Peculiarities of linear growth of the melanin-containing fungi Cladosporium sphaerospermum Penz. and Alternaria alternata (Fr.) Keissler. Mikrobiol. Z..

[318] Dadachova E (2007). Ionizing radiation changes the electronic properties of melanin and enhances the growth of melanized fungi. PLoS ONE.

[319] Dadachova E (2008). The radioprotective properties of fungal melanin are a function of its chemical composition, stable radical presence and spatial arrangement. Pigment Cell Melanoma Res..

[320] Cordero RJB (2017). Melanin for space travel radioprotection. Environ. Microbiol..

[321] Robinson CH (2001). Cold adaptation in Arctic and Antarctic fungi. New Phytol..

[322] Dadachova E (2007). The radioprotective properties of fungal melanin are a function of its chemical composition, stable radical presence and spatial arrangement. Pigment Cell Amp. Melanoma Res..

[323] Eisenman HC (2005). Microstructure of cell wall-associated melanin in the human pathogenic fungus Cryptococcus neoformans. Biochemistry.

[324] Schultzhaus Z (2020). The response of the melanized yeast Exophiala dermatitidis to gamma radiation exposure. Environ. Microbiol..

[325] Wong HJ, Mohamad-Fauzi N, Rizman-Idid M, Convey P, Alias SA (2019). Protective mechanisms and responses of micro-fungi towards ultraviolet-induced cellular damage. Polar Sci..

[326] Romsdahl J, Blachowicz A, Chiang Y-M, Venkateswaran K, Wang CCC (2020). Metabolomic analysis of Aspergillus niger isolated from the International Space Station reveals enhanced production levels of the antioxidant Pyranonigrin A. Front. Microbiol..

[327] Malo ME (2021). Transcriptomic and genomic changes associated with radioadaptation in Exophiala dermatitidis. Comput. Struct. Biotechnol. J..

[328] Cortesão M (2021). MARSBOx: fungal and bacterial endurance from a balloon-flown analog mission in the stratosphere. Front. Microbiol..

[329] Zhdanova, N. et al. Tropism of soil micromycetes under the influence of ionizing radiation. *Mikologiya I Fitopatologiya* **28**, (1994).

[330] Averesch NJH, Shunk GK, Kern C (2022). Cultivation of the Dematiaceous Fungus Cladosporium sphaerospermum Aboard the International Space Station and Effects of Ionizing Radiation. Front. Microbiol..

[331] Almpani-Lekka D, Pfeiffer S, Schmidts C, Seo S (2021). A review on architecture with fungal biomaterials: the desired and the feasible. Fungal Biol. Biotechnol..

[332] Cerimi K, Akkaya KC, Pohl C, Schmidt B, Neubauer P (2019). Fungi as source for new bio-based materials: a patent review. Fungal Biol. Biotechnol..

[333] Yang L, Park D, Qin Z (2021). Material function of mycelium-based bio-composite: a review. Front. Mater..

[334] Haneef M (2017). Advanced materials from fungal mycelium: fabrication and tuning of physical properties. Sci. Rep..

[335] Ziegler AR, Bajwa SG, Holt GA, McIntyre G, Bajwa DS (2016). Evaluation of physico-mechanical properties of mycelium reinforced green biocomposites made from cellulosic fibers. Appl. Eng. Agric..

[336] Sun W, Tajvidi M, Hunt CG, McIntyre G, Gardner DJ (2019). Fully bio-based hybrid composites made of wood, fungal mycelium and cellulose nanofibrils. Sci. Rep..

[337] Lim S (2021). Identification of the pigment and its role in UV resistance in Paecilomyces variotii, a Chernobyl isolate, using genetic manipulation strategies. Fungal Genet. Biol..

[338] Roberts AD (2021). Blood, sweat, and tears: extraterrestrial regolith biocomposites with in vivo binders. Mater. Today Bio.

[339] Sharma A, Chaudhuri TK (2017). Revisiting *Escherichia coli* as microbial factory for enhanced production of human serum albumin. Micro. Cell Fact..

[340] Roberts AD (2020). Non-covalent protein-based adhesives for transparent substrates bovine serum albumin vs. recombinant spider silk. Mater. Today Bio.

[341] Mujah D, Shahin MA, Cheng L (2016). State-of-the-art review of biocementation by microbially induced calcite precipitation (MICP) for soil stabilization. Geomicrobiol. J..

[342] Bang SS, Galinat JK, Ramakrishnan V (2001). Calcite precipitation induced by polyurethane-immobilized Bacillus pasteurii. Enzym. Micro. Technol..

[343] Stocks-Fischer S, Galinat JK, Bang SS (1999). Microbiological precipitation of CaCO3. Soil Biol. Biochem..

[344] Rahbar N (2020). Extending the life of self-healing structural. Mater. Matter.

[345] Zhu T, Paulo C, Merroun ML, Dittrich M (2015). Potential application of biomineralization by Synechococcus PCC8806 for concrete restoration. Ecol. Eng..

[346] M KS (2021). Evaluation of crack healing potential of cement mortar incorporated with blue-green microalgae. J. Build. Eng..

[347] Seifan M, Samani AK, Berenjian A (2016). Induced calcium carbonate precipitation using Bacillus species. Appl. Microbiol. Biotechnol..

[348] Mondal S, Das P, Datta P (2020). Deinococcus radiodurans: a novel bacterium for crack remediation of concrete with special applicability to low-temperature conditions. Cem. Concr. Compos.

[349] Ramachandran SK, Ramakrishnan V, Bang SS (2001). Remediation of Concrete Using Microorganisms. ACI Mater. J..

[350] Jonkers HM, Thijssen A, Muyzer G, Copuroglu O, Schlangen E (2010). Application of bacteria as self-healing agent for the development of sustainable concrete. Ecol. Eng..

[351] Barabesi C (2007). Bacillus subtilis gene cluster involved in calcium carbonate biomineralization. J. Bacteriol..

[352] Heveran CM (2020). Biomineralization and successive regeneration of engineered living building materials. Matter.

[353] Qiu J (2021). Engineering living building materials for enhanced bacterial viability and mechanical properties. iScience.

[354] Li J (2014). Genomic and transcriptomic analysis of NDM-1 Klebsiella pneumoniae in spaceflight reveal mechanisms underlying environmental adaptability. Sci. Rep..

[355] Crabbé A (2011). Transcriptional and proteomic responses of Pseudomonas aeruginosa PAO1 to spaceflight conditions involve Hfq regulation and reveal a role for oxygen. Appl. Environ. Microbiol..

[356] Nickerson CA (2000). Microgravity as a novel environmental signal affecting Salmonella enterica serovar typhimurium virulence. Infect. Immun..

[357] Wilson JW (2007). Space flight alters bacterial gene expression and virulence and reveals a role for global regulator Hfq. Proc. Natl Acad. Sci. USA.

[358] Gilbert R (2020). Spaceflight and simulated microgravity conditions increase virulence of Serratia marcescens in the Drosophila melanogaster infection model. NPJ Microgravity.

[359] Chopra V (2006). Alterations in the virulence potential of enteric pathogens and bacterial host cell interactions under simulated microgravity conditions. J. Toxicol. Environ. Health A.

[360] Knox, B. P. et al. Characterization of Aspergillus fumigatus isolates from air and surfaces of the international space station. *mSphere* **1**, (2016). 10.1128/mSphere.00227-16 PMC5082629 27830189

[361] Crabbé A (2013). Spaceflight enhances cell aggregation and random budding in Candida albicans. PLoS ONE.

[362] Wilson JW (2002). Microarray analysis identifies Salmonella genes belonging to the low-shear modeled microgravity regulon. Proc. Natl Acad. Sci. USA.

[363] Crabbé A (2010). Response of Pseudomonas aeruginosa PAO1 to low shear modelled microgravity involves AlgU regulation. Environ. Microbiol..

[364] Castro SL, Nelman-Gonzalez M, Nickerson CA, Ott CM (2011). Induction of attachment-independent biofilm formation and repression of hfq expression by low-fluid-shear culture of Staphylococcus aureus. Appl. Environ. Microbiol..

[365] Lawal A, Jejelowo OA, Rosenzweig JA (2010). The effects of low-shear mechanical stress on yersinia pestis virulence. Astrobiology.

[366] Rosado H, Doyle M, Hinds J, Taylor PW (2010). Low-shear modelled microgravity alters expression of virulence determinants of Staphylococcus aureus. Acta Astronaut..

[367] Timmery S, Hu X, Mahillon J (2011). Characterization of bacilli isolated from the confined environments of the Antarctic Concordia Station and the International Space Station. Astrobiology.

[368] O’Rourke A, Lee MD, Nierman WC, Everroad RC, Dupont CL (2020). Genomic and phenotypic characterization of Burkholderia isolates from the potable water system of the International Space Station. PLoS ONE.

[369] Hammond TG (2013). Effects of microgravity on the virulence of Listeria monocytogenes, Enterococcus faecalis, Candida albicans, and methicillin-resistant Staphylococcus aureus. Astrobiology.

[370] Mora, M. et al. Space Station conditions are selective but do not alter microbial characteristics relevant to human health. *Nat. Commun.* **10**, (2019). 10.1038/s41467-019-11682-z PMC6728350 31488812

[371] Blaustein RA (2019). Pangenomic approach to understanding microbial adaptations within a model built environment, the International Space Station, relative to human hosts and soil. mSystems.

[372] Donlan RM (2002). Biofilms: microbial life on surfaces. Emerg. Infect. Dis..

[373] Reffuveille, F., Josse, J., Vallé, Q., Mongaret, C. & Gangloff, S. C. Staphylococcus aureus Biofilms and their Impact on the Medical Field. In *The Rise of Virulence and Antibiotic Resistance in Staphylococcus aureus* (InTech, 2017).

[374] Yin W, Wang Y, Liu L, He J (2019). Biofilms: the microbial protective clothing in extreme environments. Int. J. Mol. Sci..

[375] Høiby N, Bjarnsholt T, Givskov M, Molin S, Ciofu O (2010). Antibiotic resistance of bacterial biofilms. Int. J. Antimicrob. Agents.

[376] McLean RJC, Cassanto JM, Barnes MB, Koo JH (2001). Bacterial biofilm formation under microgravity conditions. FEMS Microbiol. Lett..

[377] Kim W (2013). Spaceflight promotes biofilm formation by Pseudomonas aeruginosa. PLoS ONE.

[378] Pyle, B. et al. Bacterial Growth on surfaces and in suspensions. in *Biorack on Spacehab* vol. 148 (Biological Experiments on Shuttle to Mir Missions 03, 05, and 06, European Space Agency, 1999).

[379] Acres JM, Youngapelian MJ, Nadeau J (2021). The influence of spaceflight and simulated microgravity on bacterial motility and chemotaxis. NPJ Microgravity.

[380] Beech IB, Sunner J (2004). Biocorrosion: towards understanding interactions between biofilms and metals. Curr. Opin. Biotechnol..

[381] Sielaff AC (2019). Characterization of the total and viable bacterial and fungal communities associated with the International Space Station surfaces. Microbiome.

[382] Dai X (2016). Corrosion of aluminum alloy 2024 caused by Aspergillus niger. Int. Biodeterior. Amp. Biodegrad..

[383] Rajasekar A, Ting Y-P (2010). Microbial corrosion of aluminum 2024 aeronautical alloy by hydrocarbon degrading bacteria Bacillus cereus ACE4 and Serratia marcescens ACE2. Ind. Amp. Eng. Chem. Res..

[384] Pavissich JP, Vargas IT, González B, Pastén PA, Pizarro GE (2010). Culture dependent and independent analyses of bacterial communities involved in copper plumbing corrosion. J. Appl. Microbiol..

[385] Yang S, Lin J, Lin Y (1998). Microbiologically induced corrosion of aluminum alloys in fuel-oil/aqueous system - PubMed. J. Microbiol. Immunol. Infect..

[386] Klintworth R, Reher HJ, Viktorov AN, Bohle D (1999). Biological induced corrosion of materials II: New test methods and experiences from mir station. Acta Astronaut..

[387] Novikova N (2006). Survey of environmental biocontamination on board the International Space Station. Res. Microbiol..

[388] Novikova ND (2004). Review of the knowledge of microbial contamination of the Russian Manned Spacecraft. Micro. Ecol..

[389] Bacci G (2019). Microbial community composition of water samples stored inside the International Space Station. Res. Microbiol..

[390] Landry KS, Morey JM, Bharat B, Haney NM, Panesar SS (2020). Biofilms impacts on human health and its relevance to space travel. Microorganisms.

[391] Zea L (2018). Design of a spaceflight biofilm experiment. Acta Astronaut..

[392] Yang J (2021). Longitudinal characterization of multispecies microbial populations recovered from spaceflight potable water. npj Biofilms Microbiomes.

[393] Carter, D., Wilson, L. & Orozco, N. Status of ISS Water Management and Recovery. in *41st International Conference on Environmental Systems* (American Institute of Aeronautics and Astronautics, 2011).

[394] Zea L (2020). Potential biofilm control strategies for extended spaceflight missions. Biofilm.

[395] Tirumalai MR (2017). The adaptation of *Escherichia coli* cells grown in simulated microgravity for an extended period is both phenotypic and genomic. NPJ Microgravity.

[396] Panitz C, Frösler J, Wingender J, Flemming H-C, Rettberg P (2019). Tolerances of Deinococcus geothermalis biofilms and planktonic cells exposed to space and simulated martian conditions in low earth orbit for almost two years. Astrobiology.

[397] Flemming H-C (2016). Biofilms: an emergent form of bacterial life. Nat. Rev. Microbiol..

[398] Mauclaire L, Egli M (2010). Effect of simulated microgravity on growth and production of exopolymeric substances of Micrococcus luteus space and earth isolates. FEMS Immunol. Amp. Med. Microbiol..

[399] Guo Y (2015). Effects of space environment on genome, transcriptome, and proteome of Klebsiella pneumoniae. Arch. Med. Res..

[400] Muhammad MH (2020). Beyond risk: bacterial biofilms and their regulating approaches. Front. Microbiol..

[401] Ichikawa K, Nakamura HK, Ogawa N, Sakimura T, Kuroda M (1999). R&D of long-term life support system by using electrochemically activated biofilm reactor of aquatic animals for space examinations. Biol. Sci. Space.

[402] BfS - Biological dosimetry following radiation exposure - Biological dosimetry following radiation exposure. https://www.bfs.de/EN/topics/ion/service/dosimetry/biological-dosimetry/biological-dosimetry.html.

[403] Bérces A (1999). Biological UV dosimeters in the assessment of the biological hazard from environmental radiation. J. Photochem. Photobio. B.

[404] Rettberg P, Horneck G, Zittermann A, Heer M (1998). Biological dosimetry to determine the UV radiation climate inside the MIR station and its role in vitamin D biosynthesis. Adv. Space Res..

[405] Soucy SM, Huang J, Gogarten JP (2015). Horizontal gene transfer: building the web of life. Nat. Rev. Genet..

[406] Emamalipour M (2020). Horizontal gene transfer: from evolutionary flexibility to disease progression. Front. Cell Dev. Biol..

[407] Frost LS, Leplae R, Summers AO, Toussaint A (2005). Mobile genetic elements: the agents of open source evolution. Nat. Rev. Microbiol..

[408] Grüll, M. P., Mulligan, M. E. & Lang, A. S. Small extracellular particles with big potential for horizontal gene transfer: membrane vesicles and gene transfer agents. *FEMS Microbiol. Lett.* **365**, (2018). 10.1093/femsle/fny192 30085064

[409] Brooks AN, Turkarslan S, Beer KD, Lo FY, Baliga NS (2010). Adaptation of cells to new environments. WIREs Syst. Biol. Med..

[410] Roberts AP, Kreth J (2014). The impact of horizontal gene transfer on the adaptive ability of the human oral microbiome. Front. Cell Infect. Microbiol..

[411] Koonin EV, Makarova KS, Aravind L (2001). Horizontal gene transfer in prokaryotes: quantification and classification. Annu. Rev. Microbiol..

[412] Yamaguchi N (2014). Microbial monitoring of crewed habitats in space current status and future perspectives. Microbes Environ..

[413] Ciferri O, Tiboni O, Pasquale G, di, Orlandoni AM, Marchesi ML (1986). Effects of microgravity on genetic recombination in *Escherichia coli*. Naturwissenschaften.

[414] Juergensmeyer M, Juergensmeyer E, Guikema J (1995). Plasmid acquisition in microgravity. J. Gravit. Physiol..

[415] Boever Pde (2007). Conjugation-mediated plasmid exchange between bacteria grown under space flight conditions. Microgravity Sci. Technol..

[416] Kohler V, Keller W, Grohmann E (2019). Regulation of Gram-positive conjugation. Front. Microbiol..

[417] Beuls E (2009). Bacillus thuringiensis conjugation in simulated microgravity. Astrobiology.

[418] Urbaniak C, Grams T, Mason CE, Venkateswaran K (2021). Simulated microgravity promotes horizontal gene transfer of antimicrobial resistance genes between bacterial genera in the absence of antibiotic selective pressure. Life.

[419] Jeong H, Arif B, Caetano-Anollés G, Kim KM, Nasir A (2019). Horizontal gene transfer in human-associated microorganisms inferred by phylogenetic reconstruction and reconciliation. Sci. Rep..

[420] Najar-Peerayeh S, Moghadas AJ, Behmanesh M (2014). Antibiotic susceptibility and mecA frequency in Staphylococcus epidermidis, isolated from intensive care unit patients. Jundishapur J. Microbiol..

[421] Bloemendaal ALA, Brouwer EC, Fluit AC (2010). Methicillin resistance transfer from Staphylocccus epidermidis to methicillin-susceptible Staphylococcus aureus in a patient during antibiotic therapy. PLoS ONE.

[422] Massella E (2021). Antimicrobial resistance profile and ExPEC virulence potential in commensal *Escherichia coli* of multiple sources. Antibiotics.

[423] Floreani A, Leung PSC, Gershwin ME (2015). Environmental basis of autoimmunity. Clin. Rev. Allergy Amp. Immunol..

[424] Molina V, Shoenfeld Y (2005). Infection, vaccines and other environmental triggers of autoimmunity. Autoimmunity.

[425] Andersson DI, Hughes D (2010). Antibiotic resistance and its cost: is it possible to reverse resistance?. Nat. Rev. Microbiol..

[426] Heuer H, Schmitt H, Smalla K (2011). Antibiotic resistance gene spread due to manure application on agricultural fields. Curr. Opin. Microbiol..

[427] Nikaido H (2009). Multidrug resistance in bacteria. Annu. Rev. Biochem..

[428] Allen HK (2010). Call of the wild: antibiotic resistance genes in natural environments. Nat. Rev. Microbiol..

[429] Schiwon K (2013). Comparison of antibiotic resistance, biofilm formation and conjugative transfer of Staphylococcus and Enterococcus Isolates from International Space Station and Antarctic Research Station Concordia. Micro. Ecol..

[430] Urbaniak C (2018). Detection of antimicrobial resistance genes associated with the International Space Station environmental surfaces. Sci. Rep..

[431] Will WR, Frost LS (2006). Hfq is a regulator of F-plasmid TraJ and TraM synthesis in *Escherichia coli*. J. Bacteriol..

[432] Lerner, A., Matthias, T. & Aminov, R. Potential effects of horizontal gene exchange in the human gut. *Front. Immunol.* **8**, (2017). 10.3389/fimmu.2017.01630 PMC5711824 29230215

[433] Siddiqui R, Akbar N, Khan NA (2020). Gut microbiome and human health under the space environment. J. Appl. Microbiol..

[434] Groussin M (2021). Elevated rates of horizontal gene transfer in the industrialized human microbiome. Cell.

[435] Ghigo J-M (2001). Natural conjugative plasmids induce bacterial biofilm development. Nature.

[436] Reisner A, Haagensen JAJ, Schembri MA, Zechner EL, Molin S (2003). Development and maturation of *Escherichia coli* K-12 biofilms. Mol. Microbiol..

[437] Reisner A, Höller BM, Molin S, Zechner EL (2006). Synergistic effects in mixed *Escherichia coli* biofilms: conjugative plasmid transfer drives biofilm expansion. J. Bacteriol..

[438] Gama JA (2020). Dominance between plasmids determines the extent of biofilm formation. Front. Microbiol..

[439] Burmølle M, Bahl MI, Jensen LB, Sørensen SJ, Hansen LH (2008). Type 3 fimbriae, encoded by the conjugative plasmid pOLA52, enhance biofilm formation and transfer frequencies in Enterobacteriaceae strains. Microbiol. (N. Y).

[440] Schroll C, Barken KB, Krogfelt KA, Struve C (2010). Role of type 1 and type 3 fimbriae in Klebsiella pneumoniae biofilm formation. BMC Microbiol..

[441] Vaishampayan A, Grohmann E (2019). Multi-resistant biofilm-forming pathogens on the International Space Station. J. Biosci..

[442] Ong C-LY (2008). Identification of type 3 fimbriae in uropathogenic *Escherichia coli* reveals a role in biofilm formation. J. Bacteriol..

[443] Bai P, Li Y, Xu H (2022). Markedly decreased growth rate and biofilm formation ability of Acinetobacter schindleri after a long-duration (64 days) spaceflight. Eur. Rev. Med. Pharmacol. Sci..

[444] Zhang B, Bai P, Wang D (2022). Growth behavior and transcriptome profile analysis of Proteus Mirabilis strain under long- versus short-term simulated microgravity environment. Pol. J. Microbiol..

[445] Hausner M, Wuertz S (1999). High rates of conjugation in bacterial biofilms as determined by quantitative in situ analysis. Appl. Environ. Microbiol..

[446] Lécuyer F (2018). Biofilm formation drives transfer of the conjugative element ICE Bs1 in Bacillus subtilis. mSphere.

[447] Uruén C, Chopo-Escuin G, Tommassen J, Mainar-Jaime RC, Arenas J (2020). Biofilms as promoters of bacterial antibiotic resistance and tolerance. Antibiotics.

[448] Savage VJ, Chopra I, ONeill AJ (2013). Staphylococcus aureus biofilms promote horizontal transfer of antibiotic resistance. Antimicrob. Agents Chemother..

[449] Trieu-Cuot P, Carlier C, Martin P, Courvalin P (1987). Plasmid transfer by conjugation from *Escherichia coli* to Gram-positive bacteria. FEMS Microbiol. Lett..

[450] Mazodier P, Petter R, Thompson C (1989). Intergeneric conjugation between *Escherichia coli* and Streptomyces species. J. Bacteriol..

[451] Hamilton TA (2019). Efficient inter-species conjugative transfer of a CRISPR nuclease for targeted bacterial killing. Nat. Commun..

[452] Dominguez W, O’Sullivan DJ (2013). Developing an efficient and reproducible conjugation-based gene transfer system for bifidobacteria. Microbiology.

[453] Samperio S (2021). Conjugative DNA transfer from *E. coli* to transformation-resistant Lactobacilli. Front. Microbiol..

[454] Brumwell, S. L., Belois, K. D., van, Giguere, D. J., Edgell, D. R. & Karas, B. J. Conjugation-based genome engineering in Deinococcus radiodurans. 10.1101/2021.10.13.464295 (2021). 10.1021/acssynbio.1c00524 PMC8939323 35254818

[455] Heinemann JA, Sprague GF (1989). Bacterial conjugative plasmids mobilize DNA transfer between bacteria and yeast. Nature.

[456] Reuter, A. et al. Targeted-antibacterial-plasmids (TAPs) combining conjugation and CRISPR/Cas systems achieve strain-specific antibacterial activity. 10.1101/2020.10.12.335968 (2020). 10.1093/nar/gkab126 PMC8034655 33660775

[457] Kiga K (2020). Development of CRISPR-Cas13a-based antimicrobials capable of sequence-specific killing of target bacteria. Nat. Commun..

[458] Price, V. J. et al. Enterococcus faecalis CRISPR-Cas is a robust barrier to conjugative antibiotic resistance dissemination in the murine intestine. 10.1101/312751 (2018). 10.1128/mSphere.00464-19 PMC6656873 31341074

[459] Zhou Y (2020). The type I-E CRISPR-Cas system influences the acquisition of blaKPC-IncF plasmid in Klebsiella pneumonia. Emerg. Microbes Infect..

[460] Singh NK, Wood JM, Karouia F, Venkateswaran K (2018). Succession and persistence of microbial communities and antimicrobial resistance genes associated with International Space Station environmental surfaces. Microbiome.

[461] Bryan NC (2021). Genomic and functional characterization of Enterococcus faecalis isolates recovered from the International Space Station and their potential for pathogenicity. Front. Microbiol..

[462] Marraffini LA, Sontheimer EJ (2008). CRISPR interference limits horizontal gene transfer in Staphylococci by targeting DNA. Science.

[463] Kaminski MM, Abudayyeh OO, Gootenberg JS, Zhang F, Collins JJ (2021). CRISPR-based diagnostics. Nat. Biomed. Eng..

[464] Rauch JN (2021). A scalable, easy-to-deploy protocol for Cas13-based detection of SARS-CoV-2 genetic material. J. Clin. Microbiol..

[465] Broughton JP (2020). CRISPR–Cas12-based detection of SARS-CoV-2. Nat. Biotechnol..

[466] Stahl-Rommel S (2021). A CRISPR-based assay for the study of eukaryotic DNA repair onboard the International Space Station. PLoS ONE.

[467] Clark, R. L. et al. Design of synthetic human gut microbiome assembly and function. 10.1101/2020.08.19.241315 (2020).

[468] Wu H, Moser C, Wang H-Z, Høiby N, Song Z-J (2014). Strategies for combating bacterial biofilm infections. Int. J. Oral. Sci..

[469] Buchovec I, Gricajeva A, Kalėdienė L, Vitta P (2020). Antimicrobial photoinactivation approach based on natural agents for control of bacteria biofilms in Spacecraft. Int. J. Mol. Sci..

[470] Hahn C (2017). Pure and oxidized copper materials as potential antimicrobial surfaces for spaceflight activities. Astrobiology.

[471] Siems K (2022). Testing laser-structured antimicrobial surfaces under space conditions: the design of the ISS experiment BIOFILMS. Front. Space Technol..

[472] Sobisch L-Y (2019). Biofilm forming antibiotic resistant Gram-positive pathogens isolated from surfaces on the International Space Station. Front. Microbiol..

[473] Pipiya SO (2020). Engineering artificial biodiversity of lantibiotics to expand chemical space of DNA-encoded antibiotics. Biochemistry.

[474] Walsh DJ, Livinghouse T, Goeres DM, Mettler M, Stewart PS (2019). Antimicrobial activity of naturally occurring phenols and derivatives against biofilm and Planktonic bacteria. Front. Chem..

[475] Francolini I, Vuotto C, Piozzi A, Donelli G (2017). Antifouling and antimicrobial biomaterials: an overview. APMIS.

[476] Goodband SJ, Armstrong S, Kusumaatmaja H, Vötchovsky K (2020). Effect of ageing on the structure and properties of model liquid-infused surfaces. Langmuir.

